# Cost-effectiveness analysis of typhoid conjugate vaccines in five endemic low- and middle-income settings

**DOI:** 10.1016/j.vaccine.2017.05.001

**Published:** 2017-06-14

**Authors:** Marina Antillón, Joke Bilcke, A. David Paltiel, Virginia E. Pitzer

**Affiliations:** a Department of Epidemiology of Microbial Diseases, Yale School of Public Health, New Haven, CT 06520-8034, USA; b Center for Health Economics Research and Modeling Infectious Diseases, University of Antwerp, Belgium; c Department of Health Policy and Management, Yale School of Public Health, New Haven, CT 06520-8034, USA

**Keywords:** Typhoid, Conjugate vaccines, Cost-effectiveness studies, Low- and middle-income countries

## Abstract

**Background:**

Typhoid fever remains endemic in low- and middle-income countries. Programmatic use of existing vaccines is limited, but upcoming typhoid conjugate vaccines (TCVs) could warrant wider use. We evaluated the cost-effectiveness of five TCV delivery strategies in three urban areas (Delhi and Kolkata, India and Nairobi, Kenya) and two rural settings (Lwak, Kenya and Dong Thap, Vietnam) with varying incidence.

**Methods and findings:**

We evaluated routine infant vaccination with and without catch-up campaigns among older individuals. We used a dynamic model of typhoid transmission to simulate cases, hospitalizations, deaths, disability-adjusted life-years (DALY) lost, treatment and intervention costs. We estimated cost-effectiveness (in terms of cost in international dollars (I$) per DALY averted) from the healthcare payer perspective, and assessed how it was influenced by uncertain model parameters. Compared to no vaccination, routine infant vaccination at I$1/dose was cost-saving in Delhi and Dong Thap, “very cost-effective” in Kolkata and Nairobi, and “cost-effective” in Lwak according to World Health Organization thresholds. However, routine vaccination was not the optimal strategy compared to strategies that included a catch-up campaign, which yielded the highest probability of being cost-saving in Delhi and Dong Thap and were most likely to provide a return on investment above a willingness-to-pay threshold of I$1440 in Kolkata, I$2300 in Nairobi, and I$5360 in Lwak. Vaccine price impacted the optimal strategy, and the number of doses required and rate of hospitalization were the primary sources of uncertainty.

**Conclusion:**

Routine vaccination with TCV would be cost-effective in most settings, and additional one-time catch-up campaigns would also be economically justified.

## 1 Introduction

Between 11.9–26.9 million cases of typhoid fever occur each year in low- and middle-income countries (LMICs) [1], [2], [48], [49]. Symptoms include fever, abdominal pain, and nausea, which last between one to four weeks, and 1–2% of hospitalized cases result in death [3], [4]. Improved sanitation contributed to the sharp decline of typhoid fever in industrialized countries during the early 20th century [4], [5], but such infrastructure is slow to materialize in places where the disease remains endemic and antibiotic-resistance is on the rise [4], [6]. Vaccination may prove a timely measure to abate the burden of disease.

Although the World Health Organization (WHO) recommends the Vi-polysaccharide (ViPS) and the live-oral Ty21a vaccines in populations at high risk for typhoid fever (including children 2–15 years old), vaccine introduction has been limited [7], [8]. Newly-developed typhoid conjugate vaccines (TCVs) may prompt greater programmatic use [8], [9]. Whereas existing vaccines confer 50–70% protection for 3–5 years in individuals over 2 years of age, TCVs have a higher efficacy, longer duration of protection, and are safe and immunogenic in children as young as 6 months old, making TCVs compatible with existing infant immunization programs [10], [11], [12], [13], [14]. In light of the recent licensure of TCVs in India and the anticipated licensure in other countries, the WHO will soon be updating their recommendations for typhoid vaccine use [15], [16].

The optimal delivery strategy, economic resource implications, and cost-effectiveness of TCVs remain unknown. We sought to inform the decision-making process surrounding TCV use by examining whether a variety of vaccine delivery strategies aimed at children and adults might represent a comparatively efficient use of scarce resources.

## 2 Methods

We selected five settings that are distinct in their typhoid burden and costs of illness and vaccine delivery to carry out a comprehensive cost-effectiveness analysis that illustrates the interplay of various epidemiological and economic factors. Kolkata, India and Nairobi, Kenya represent high-incidence urban settings with moderate and low costs of illness, respectively, whereas Delhi, India represents an urban setting with very high incidence and a high cost of illness. Dong Thap, Vietnam represents a high-incidence rural setting with moderate cost of illness and Lwak, Kenya represents a medium-incidence rural setting with a low cost of illness (S1 Table) [17], [18], [19], [20], [21], [22], [23], [24].

We simulated five TCV delivery strategies using a dynamic model of typhoid transmission in a theoretical open cohort of 100,000 people (with births and deaths): (I) routine vaccination at 9 months of age; and routine vaccination at 9 months plus a one-time catch-up campaign among individuals (II) 9 months to 5 years old, (III) 9 months to 15 years old, (IV) 9 months to 25 years old, (V) all ages ≥9 months. Because incidence often peaks in school-age children or young adults in low- and medium-incidence settings, we postulated that one-time campaigns could prove cost-effective in these settings [2], [17].

Because vaccine prices have yet to be negotiated by the appropriate stakeholders, we assumed a price of 1 international dollar (I$) per dose in a single-dose schedule; we examined alternative pricing and dosing schedules in scenario analyses. We assumed 80% coverage for routine vaccination and 70% coverage for campaigns.

The null comparator is a scenario with no vaccination, which is the current strategy in most LMICs [8], [9], [15]. Our main outcome was the incremental cost-effectiveness ratio (ICER), defined as the cost (in international dollars) per disability-adjusted life-years (DALYs) averted by each strategy over a 10-year time horizon.

The analysis was conducted in accordance with the recommendations of the Bill and Melinda Gates Foundation’s (BMGF) reference case and WHO guidelines [25], [26], [27]. We adopted the perspective of the healthcare payer, therefore considering only the DALYs lost by care-seeking individuals and the direct treatment and vaccination costs accrued by the healthcare system [25], [26].

### 2.1 Model structure

**Fig. 1:**
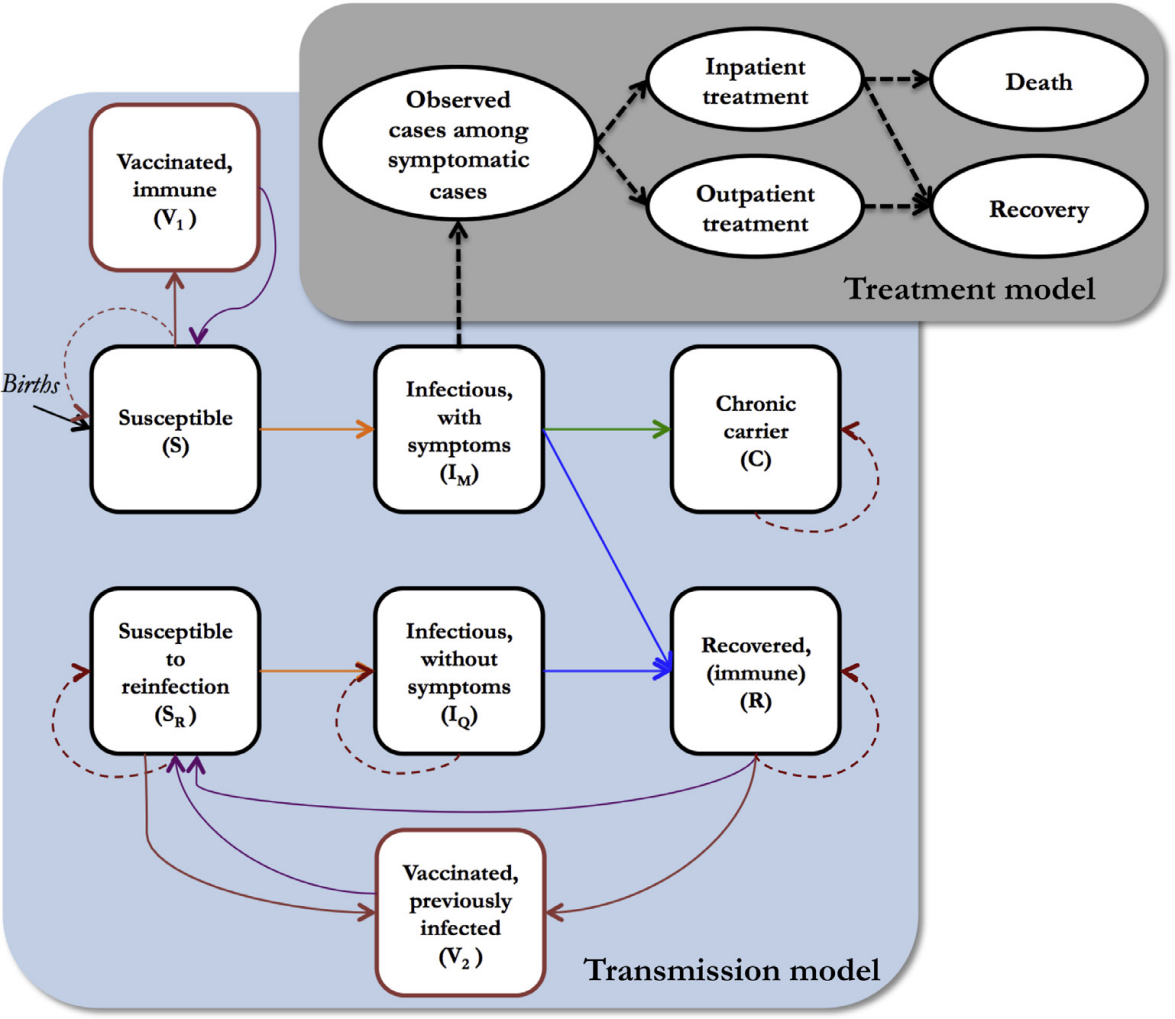
Transmission and treatment model. The transmission model (black squares) includes: two susceptible classes—one for individuals who have never been previously infected and another for individuals whose immunity to reinfection has waned; two infectious classes—one for primary infections and another for subsequent infections, which we assume are subclinical; a recovered class, which is temporarily immune to reinfection; and a class of chronic carriers, who are assumed to remain infectious until death. We also model two vaccinated classes (red boxes)—one for individuals who have been successfully immunized and are protected from symptomatic infection, and another for individuals who had been previously infected and who are only protected from asymptomatic infection. Orange lines depict the infection process, blue lines depict the recovery process, green lines depict the process by which individuals become chronic carriers, purple lines depict waning immunity, and red lines signify the vaccination process. The dashed red lines correspond to individuals who do not respond to vaccination. The treatment model depicts a probability tree of treatment outcomes (black ovals). The dashed black lines represent probabilistic binomial samples. (For interpretation of the references to colour in this figure legend, the reader is referred to the web version of this article.)

We modified an existing age-stratified compartmental model of typhoid transmission (“transmission model”) and added a probability model describing treatment outcomes (“treatment model”) (Fig. 1) [28]. Explicitly modeling the transmission dynamics allowed us to fully account for the decreased risk of infection that vaccination may confer on the population (herd immunity). We modeled each population using setting-specific transmission parameters, which we estimated by fitting the model to the adjusted age-specific incidence in each setting using a Hamiltonian Monte Carlo sampling algorithm (see S1 Appendix §1-3) [29].

The probability of receiving inpatient or outpatient care was parameterized using published data specific to each site; the costs and duration of disease were contingent on the type of care received [10], [17], [18], [19], [20], [22], [23], [24]. DALYs were calculated as the sum of years lost to morbidity and death (discounted for severity and time) (see S1 Appendix §5) [30]. We assumed neither sanitation improvements nor enhanced capacity to treat or isolate cases would occur over the time horizon of our analysis.

### 2.2 Data

We collated data on disease incidence, progression, and mortality from the published literature to parameterize probability distributions describing uncertainty around the transmission, natural history, and costs associated with treatment of typhoid fever and vaccination campaigns (Table 1; see S1 Appendix §1, §4, and §6). We used efficacy data from a clinical trial of the two-dose Vi-rEPA conjugate vaccine to inform the probability of vaccine protection and duration of immunity (see S1 Appendix §4) [10]. The leading TCV candidate (Typbar-TCV, Bharat Biotech) was proven effective on the basis of serological surrogates of efficacy, suggesting that a single dose would provide comparable protection to Vi-rEPA [13], [14], [15]. All of the data we used to parameterize the costs of treatment and vaccination came from micro-costing studies, except for vaccine administrative costs in India, which came from the country Multi-Year Plan (see S1 Appendix §6) [31], [32], [33]. Because we did not have data itemized by each of the components of operational vaccination costs (e.g. storage, administration equipment and personnel, etc.), we assumed full operational costs rather than incremental costs; at worst, this assumption would bias our analysis against vaccine adoption.

**Table 1:** Input parameters for treatment outcomes and treatment and intervention costs. The sources for the parameters are detailed in the Appendix.

Parameter	Kolkata	Delhi	Dong Thap	Nairobi	Lwak
*Treatment outcomes*					
Duration of disease in weeks: Inpatient (range)	2–4	2–4	2–4	2–4	2–4
Relative duration of disease: Outpatient vs Inpatient (range)	0.25–0.75	0.25–0.75	0.25–0.75	0.25–0.75	0.25–0.75
Disability weight	0.13 (0.025)	0.13 (0.025)	0.13 (0.025)	0.13 (0.025)	0.13 (0.025)
Probability of hospitalization	0.03 (0.015)	0.09 (0.035)	0.33 (0.0575)	0.02 (0.0125)	0.23 (0.08)
Probability of death among inpatients	0.016 (0.004)	0.016 (0.004)	0.016 (0.004)	0.016 (0.004)	0.016 (0.004)

*Treatment and intervention costs (2015 I$)*					
Outpatient treatment costs	18.69 (1.27)	222.12 (17.48)	10.70 (4.09)	4.78 (1.88)	4.78 (1.88)
Inpatient treatment costs	928.43 (101.45)	4840.50 (755.17)	1241.32 (475)	103.87 (28.72)	103.87 (28.72)
Vaccine supplies	0.17 (0.06)	0.17 (0.06)	0.14 (0.05)	0.19 (0.07)	0.19 (0.07)
Operational costs - routine	3.55 (1.36)	3.55 (1.36)	8.33 (3.19)	3.61 (0.56)	3.61 (0.56)
Operational costs - campaign	1.67 (0.64)	1.67 (0.64)	9.02 (3.45)	3.61 (0.56)	3.61 (0.56)

*Fixed parameters*					
GDP per capita, I$	6088.60	6088.60	6022.60	3082.50	3082.50
Life expectancy (years)	68	68	75.6	62	62
Discount rate (% per year)	3	3	3	3	3
Vaccine wastage (%)	15	15	15	15	15

Notes:

1. Mean (standard error) are presented for uncertain parameters, unless otherwise noted.

2. Abbreviations: I$, international dollars: GDP, gross domestic product.

3. Whenever the literature did not report information on the uncertainty of one of our input parameters (e.g. a standard error around the mean inpatient cost), we assumed a wide uncertainty range, with the lower and upper limit 75% below/above the mean.

Uncertainty distributions were assigned to each input parameter according to Briggs et al. 2006 [34]. We adjusted all costs to 2015 local currency units and converted them to international dollars [25], [26]. Further details on how we resolved inconsistencies or shortcomings in the data are found in S1 Appendix §6.

### 2.3 Cost effectiveness analysis

For each setting and intervention, we estimated the ICER at a point-estimate of the transmission, treatment, and economic model parameters. As per WHO guidelines, we defined an intervention to be “very cost-effective” if the ICER was less than the per capita gross domestic product (GDP) of the country, and an intervention was “cost-effective” if the ICER was less than three times the per capita GDP.

We then adopted a net benefits framework to evaluate the probability that each strategy was optimal across a range of willingness-to-pay (WTP) thresholds while accounting for parameter uncertainty [34], [35]. Net monetary benefits (NMB) are defined as the product of the WTP threshold and the DALYs averted by the intervention, minus the cost of the intervention [34], [35]. To calculate the NMB at each WTP value, we drew 1000 samples from the joint posterior distribution of model parameters. [34], [35]. First, we assessed the probability that routine vaccination alone (intervention I) was the preferred strategy (had a NMB > I$0) compared to the status quo (no vaccination) at WTP thresholds of $0–$20,000 per DALY averted, which spans the range of zero to three times the per capita GDP for all countries in our analysis. We then assessed the four additional strategies that included a catch-up campaign (II–V) alongside intervention I. When comparing multiple interventions, an intervention is considered optimal at a given WTP threshold with probability equal to the proportion of samples in which the intervention in question had the highest NMB [34], [35].

### 2.4 Scenario and sensitivity analysis

We performed five additional scenario analyses that evaluated the cost-effectiveness of all delivery strategies under the assumption that the cost per dose would be I$2 or I$5 (based on the costs of other Gavi-supported vaccines) and the current price of the Typbar-TCV vaccine in India (1800 INR) [15], and under the assumption that two doses (and thus two visits to the vaccination post) would be necessary in order to completely immunize children <5 years of age at I$1, I$2, or I$5/dose. Some of the TCV candidates require two doses to fully immunized children <5 years of age.

We performed sensitivity analyses to examine the robustness of our findings to parameter uncertainty. Because some of our model parameters are either not normally distributed or are correlated to other parameters, we used random forest analysis instead of the more typical ANOVA methods to estimate the relative contribution of each parameter to uncertainty in the NMB at a WTP value equivalent to the GDP per capita in each country (see S1 Appendix §7) [36], [37].

### 2.5 Role of the funding source

This study was funded by the Bill and Melinda Gates Foundation (BMGF); therefore, we conducted the cost-effectiveness analysis in accordance with the BMGF reference case. An advisory board hosted by BMGF provided input on which delivery strategies to evaluate; otherwise, the funders had no role in the study design, data collection and analysis, preparation of the manuscript, nor the decision to submit for publication.

## 3 Results

Our transmission model provided a good fit to the observed incidence of typhoid fever by age across the five settings (S5 Figure). The predicted incidence rate did not differ significantly from the observed incidence rate after adjusting for blood culture sensitivity and the observed participation rate (Table 2).

**Table 2:** Impact of various vaccination strategies against typhoid fever on typhoid disease and economic burden. Summary of model predictions in an open cohort of 100,000 people over 10 years after the beginning of the intervention.

	Kolkata	Delhi	Dong Thap	Nairobi	Lwak
*Typhoid disease and economic burden*					
Incidence (cases per 100,000 person-years)	157 (127, 190)	754 (583, 957)	196 (149, 253)	247 (208, 291)	28 (18, 42)
Adjusted incidence (per 100,000 person-years)	280 (213, 373)	2844 (2008, 4060)	534 (382, 750)	1143 (833, 1612)	125 (66, 246)
Model-predicted incidence (per 100,000 person-years)	287 (218, 383)	2153 (1723, 2306)	542 (392, 743)	880 (676, 1195)	98 (60, 164)
Total cases	2870 (2180, 3834)	21,528 (17,233, 23,059)	5420 (3917, 7434)	8798 (6759, 11,948)	976 (596, 1644)
Hospitalizations	72 (14, 202)	1930 (842, 3904)	1742 (1051, 2796)	167 (41, 457)	233 (85, 511)
Deaths	1 (0, 3)	31 (12, 68)	28 (14, 52)	3 (1, 8)	4 (1, 9)
DALYs lost	75 (21, 200)	2008 (839, 4230)	1807 (871, 3359)	178 (61, 472)	180 (59, 444)
YLD lost	10 (3, 27)	80 (27, 197)	25 (9, 59)	31 (10, 82)	4 (1, 12)
YLL lost	64 (12, 195)	1920 (750, 4164)	1780 (855, 3339)	143 (32, 438)	176 (56, 438)
Discounted DALYs lost	36 (12, 91)	840 (365, 1738)	716 (349, 1316)	92 (33, 216)	83 (27, 202)
Discounted YLD lost	9 (3, 23)	68 (23, 168)	21 (8, 51)	26 (8, 70)	4 (1, 10)
Discounted YLL lost	27 (5, 82)	765 (298, 1663)	694 (331, 1292)	61 (14, 186)	80 (25, 194)
Discounted cost of treatment (in thousands I$)	102 (53, 210)	11,579 (6803, 20,618)	1784 (680, 3961)	50 (24, 97)	23 (9, 57)

*Impact of vaccination on typhoid disease and economic burden*					
Cases averted					
Intervention I (Routine vaccination at 9m)	527 (292, 798)	8114 (5302, 10,094)	1275 (719, 1993)	3642 (2397, 5332)	254 (127, 498)
Intervention II (Routine & campaign 9m-5y)	926 (495, 1394)	10,723 (6649, 13,419)	1979 (1087, 3061)	5010 (3285, 7142)	353 (181, 663)
Intervention III (Routine & campaign 9m-15y)	1528 (843, 2234)	12,822 (7552, 16,134)	3201 (1851, 4795)	5971 (3948, 8472)	526 (289, 926)
Intervention IV (Routine & campaign 9m-25y)	1818 (1024, 2626)	13,872 (8107, 17,484)	3506 (2061, 5144)	6646 (4.456, 9342)	589 (329, 1035)
Intervention V (Routine & campaign all ages)	2128 (1298, 3012)	16,182 (8963, 19,698)	4049 (2521, 5896)	7124 (4921, 9972)	680 (391, 1206)
Hospitalizations averted					
Intervention I (Routine vaccination at 9m)	13 (3, 40)	723 (277, 1572)	409 (212, 738)	67 (16, 190)	60 (19, 141)
Intervention II (Routine & campaign 9m-5y)	22 (5, 70)	959 (361, 2095)	631 (331, 1115)	92 (22, 260)	84 (27, 193)
Intervention III (Routine & campaign 9m-15y)	37 (7, 115)	1138 (436, 2455)	1021 (545, 1725)	111 (26, 310)	124 (41, 279)
Intervention IV (Routine & campaign 9m-25y)	44 (9, 136)	1229 (461, 2656)	1120 (598, 1859)	124 (29, 339)	139 (47, 311)
Intervention V (Routine & campaign all ages)	52 (11, 159)	1425 (530, 3110)	1308 (717, 2123)	133 (32, 368)	162 (55, 365)
Deaths averted					
Intervention I (Routine vaccination at 9m)	0 (0, 1)	12 (4, 27)	7 (3, 14)	1 (0, 3)	1 (0, 2)
Intervention II (Routine & campaign 9m-5y)	0 (0, 1)	16 (5, 36)	10 (4, 21)	1 (0, 5)	1 (0, 3)
Intervention III (Routine & campaign 9m-15y)	1 (0, 2)	18 (6, 44)	16 (7, 33)	2 (0, 5)	2 (1, 5)
Intervention IV (Routine & campaign 9m-25y)	1 (0, 2)	20 (6, 47)	18 (8, 36)	2 (0, 6)	2 (1, 6)
Intervention V (Routine & campaign all ages)	1 (0, 3)	23 (7, 54)	21 (10, 41)	2 (0, 6)	3 (1, 7)
Discounted DALYs averted					
Intervention I (Routine vaccination at 9m)	6 (2, 18)	310 (118, 666)	161 (71, 338)	37 (13, 90)	22 (6, 56)
Intervention II (Routine & campaign 9m-5y)	12 (3, 32)	418 (155, 926)	258 (114, 532)	52 (18, 126)	31 (8, 79)
Intervention III (Routine & campaign 9m-15y)	19 (5, 52)	503 (184, 1126)	423 (190, 837)	62 (22, 152)	46 (13, 117)
Intervention IV (Routine & campaign 9m-25y)	23 (6, 61)	543 (199, 1226)	462 (214, 904)	70 (24, 169)	51 (15, 129)
Intervention V (Routine & campaign all ages)	27 (8, 72)	624 (229, 1410)	531 (254, 1030)	75 (26, 182)	59 (18, 143)
Averted cost of treatment (in thousands I$, discounted)					
Intervention I (Routine vaccination at 9m)	17 (9, 39)	4252 (2605, 7218)	397 (153, 843)	20 (10, 39)	6 (2, 14)
Intervention II (Routine & campaign 9m-5y)	31 (17, 65)	5777 (3526, 9524)	634 (252, 1277)	28 (14, 53)	8 (3, 19)
Intervention III (Routine & campaign 9m-15y)	54 (31, 108)	7004 (4395, 11,252)	1069 (431, 2166)	34 (18, 61)	12 (5, 28)
Intervention IV (Routine & campaign 9m-25y)	65 (38, 127)	7608 (4934, 11,794)	1175 (477, 2392)	38 (20, 68)	14 (6, 32)
Intervention V (Routine & campaign all ages)	77 (44, 145)	8918 (5801, 13,185)	1364 (551, 2736)	41 (21, 73)	16 (6, 39)
Cost of intervention (in thousands I$)					
Intervention I (Routine vaccination at 9m)	39 (23, 65)	72 (42, 121)	128 (68, 234)	107 (86, 134)	144 (115, 177)
Intervention II (Routine & campaign 9m-5y)	50 (33, 78)	91 (60, 140)	183 (111, 290)	151 (128, 179)	184 (156, 220)
Intervention III (Routine & campaign 9m-15y)	85 (61, 119)	131 (92, 186)	338 (199, 534)	219 (190, 258)	266 (227, 312)
Intervention IV (Routine & campaign 9m-25y)	120 (87, 168)	162 (115, 226)	424 (240, 695)	280 (241, 330)	317 (271, 373)
Intervention V (Routine & campaign all ages)	208 (148, 301)	241 (170, 333)	693 (380, 1179)	386 (327, 462)	421 (357, 498)
Net costs of intervention (compared to no intervention; discounted, in thousands I$)					
Intervention I (Routine vaccination at 9m)	20 (−5, 49)	−4136 (−7742, −2176)	−246 (−829, 17)	87 (57, 116)	137 (109, 173)
Intervention II (Routine & campaign 9m-5y)	19 (−23, 49)	−5631 (−10,498, −2932)	−422 (−1307, −15)	123 (86, 155)	176 (145, 212)
Intervention III (Routine & campaign 9m-15y)	31 (−41, 74)	−6736 (−12,717, −3454)	−684 (−2066, 18)	185 (140, 227)	252 (212, 301)
Intervention IV (Routine & campaign 9m-25y)	56 (−32, 114)	−7251 (−13,747, −3645)	−697 (−2147, 75)	241 (188, 295)	301 (252, 359)
Intervention V (Routine & campaign all ages)	131 (20, 229)	−8355 (−15,969, −4112)	−617 (−2302, 377)	343 (276, 422)	403 (338, 482)

*Cost-effectiveness of vaccination against typhoid fever*					
Incremental cost-effectiveness ratios (I$/DALYs averted)					
Intervention I vs. no vaccination (status quo)	3172	Cost-saving	Cost-saving	2390	6931
Comparison of five interventions (I$/DALYs averted); the optimal intervention in terms of cost-effectiveness is italicized for each setting					
Intervention I (Routine vaccination at 9m)	Dominated	Dominated	Dominated	Weakly dominated	Weakly dominated
Intervention II (Routine & campaign 9m-5y)	Weakly dominated	Dominated	Dominated	*2368*	Weakly dominated
Intervention III (Routine & campaign 9m-15y)	*1263*	Dominated	Dominated	6138	*6092*
Intervention IV (Routine & campaign 9m-25y)	6238	Dominated	*Cost-saving*	8989	9960
Intervention V (Routine & campaign all ages)	20,442	*Cost-saving*	1655	23,628	17,007

Notes:

1. Abbreviations: I$, international dollars; YLD, years lost to disability; YLL, years of life lost; DALYs, disability-adjusted life years (the sum of YLD and YLL; see S1 Appendix §5-6).

2. Median model output and 95% credible intervals are presented.

3. “Incidence” represents the crude incidence (per 100,000 person-years) observed in the study. “Adjusted incidence” represents the crude incidence in each study after adjusting for the observation process, which included adjustments for the reported proportion of patients meeting the case definition who agreed to participate in the study and had blood drawn for diagnosis and blood culture sensitivity (see S1 Appendix §2). “Model-predicted incidence” represents the incidence predicted by the dynamic model (Fig. 1).

4. Costs of treatment and of the intervention are discounted.

5. The status quo and the intervention were modeled in an open cohort, taking into account births and deaths.

6. Interventions were considered “very cost-effective” and “cost-effective” if the ICER was below one and three times the national gross domestic product (GDP) per capita (I$6088.60 in India, I$6022.60 in Vietnam, and I$3028.50 in Kenya).

The hospitalization rate had a notable impact on the DALYs lost over 10 years (Table 2). For instance, although we calculated the lowest number of cases for Lwak, the number of DALYs lost was lowest in Kolkata due to its low hospitalization rate, and by extension of our assumptions, its low mortality rate; the difference in deaths between settings (0.02% of cases in Nairobi vs 0.46% of all cases in Dong Thap) derived from the difference in hospitalization probabilities across sites, since we assumed that the probability of death was conditional on hospitalization and was equal across settings. The costs of treatment showed a different trend. Kolkata, which experienced the lowest number of DALYs lost, had higher costs of treatment than Lwak or Nairobi. Delhi had by far the highest costs, outpacing the cost of treatment in Dong Thap by nearly an order of magnitude (Table 2).

### 3.1 Vaccine impact and net costs

**Fig. 2:**
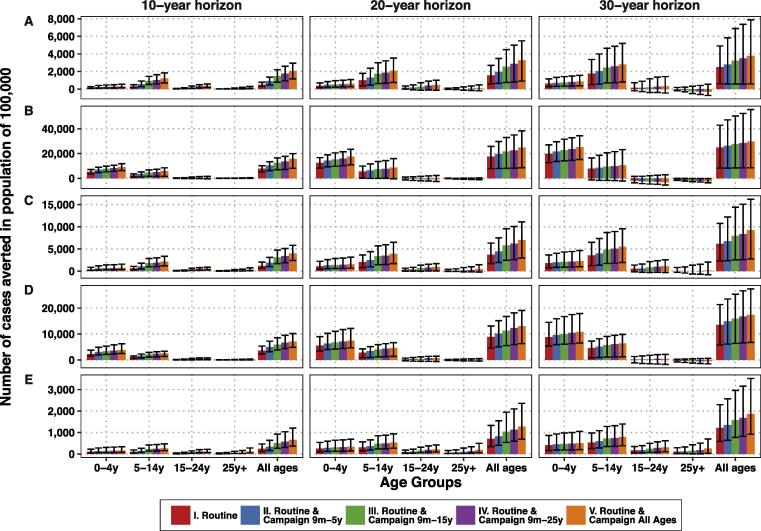
Model predictions for the cumulative number of cases averted over 10, 20, and 30 years following vaccine introduction in four age groups and in the whole population for (A) Kolkata, (B) Delhi, (C) Dong Thap, (D) Nairobi, and (E) Lwak. Note that the scale of the y-axis varies for each location. The colored bars represent the mean model predictions, while the black error bars represent the uncertainty from the transmission model parameters. (For interpretation of the references to colour in this figure legend, the reader is referred to the web version of this article.)

Our transmission model predicted a significant decrease in typhoid incidence resulting from any of the TCV delivery strategies (S6 Figure). Interventions that coupled routine vaccination with one-time catch-up campaigns yielded additional DALYs averted (Table 2). The largest decline in cases would occur in the ten years following vaccine introduction (Fig. 2). In the third decade after vaccine introduction, older age groups were predicted to experience a slight increase in typhoid incidence in some settings; this occurred because vaccination delays the time to infection but cannot completely prevent all cases. However, this did not undermine the gains from vaccination, as the overall cases averted over 30 years were still significantly greater than zero (Fig. 2).

The net costs of vaccination varied considerably among the five settings (Table 2). The costs of treatment in Delhi were high enough that the investment in vaccines was predicted to pay for itself. In Kolkata and Dong Thap, TCV introduction might save money, whereas in Nairobi and Lwak, vaccination would come at a cost.

### 3.2 Cost-effectiveness

Routine vaccination alone was predicted to be cost-saving in Delhi and Dong Thap, “very cost-effective” in Kolkata and Nairobi, and “cost-effective” in Lwak when compared to no vaccination (Table 2). In planning scenarios in which a catch-up campaign would be considered, however, routine vaccination (intervention I) was dominated in Kolkata, Delhi, and Dong Thap (Table 2).

**Fig. 3:**
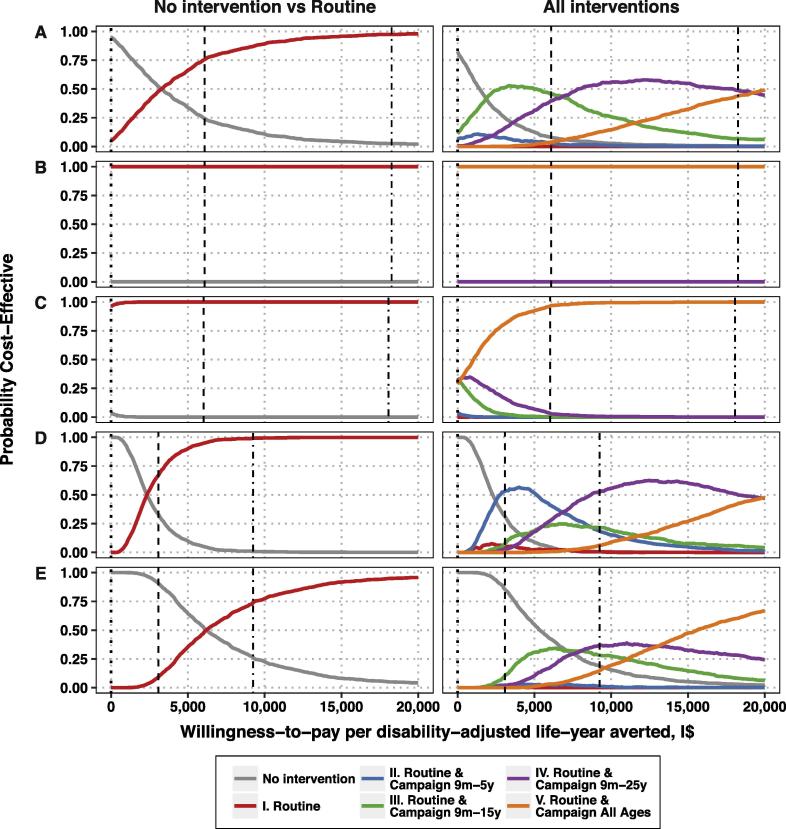
Cost-effectiveness acceptability curves for routine vaccination at 9 months of age (left) and for all five delivery strategies under consideration (right) versus no intervention for (A) Kolkata, (B) Delhi, (C) Dong Thap, (D) Nairobi, and (E) Lwak. The dotted line shows the threshold at which an intervention is considered cost-saving, while the dashed line delineates the threshold at which an intervention is considered very cost-effective and the dot-dashed line delineates the threshold at which an intervention is considered cost-effective by the WHO criteria in each country.

Accounting for parameter uncertainty, routine vaccination had a 100% and 96% chance of being cost-saving (compared to no intervention) in Delhi and Dong Thap, respectively (Fig. 3; S7 Figure). In Kolkata and Nairobi, routine vaccination was cost-effective at thresholds of more than I$3300 and I$2330 respectively—below the WHO threshold for “very cost-effective” interventions in India and Kenya. In Lwak, however, routine vaccination was optimal at thresholds of more than I$6200—above the threshold for “very cost-effective” interventions but below the threshold for “cost-effective” interventions in Kenya.

Compared to other delivery strategies, however, routine vaccination alone (intervention I) never showed the highest probability of being cost-effective (Fig. 3). In Delhi, the most ambitious intervention (V) was unequivocally the most likely to be cost-saving. In Dong Thap, interventions III–V were equally likely to be cost-saving (when WTP = I$0), and intervention V was optimal even at low WTP thresholds. In Nairobi and Kolkata, the status quo had a <50% probability of being optimal at values of $1440 and $2300, respectively, and above that the optimal intervention depended on the WTP, but routine vaccination (intervention I) was a sub-optimal strategy at all WTP values. In Lwak, the status quo had a >50% probability of being optimal at WTP values below I$5360; above this threshold, when vaccination is considered, the optimal strategy would include a catch-up campaign among children up to 15 years (intervention III) or 25 years of age (intervention IV).

### 3.3 Scenario analysis

Higher vaccine prices generally increased the WTP threshold at which interventions would become optimal except in Delhi, where the most ambitious intervention remained cost-saving (S8 Figure, S10 Figure). Routine vaccination would most likely be “cost-effective” at I$5/dose in Kolkata and Nairobi, but not in Lwak (S8 Figure). However, routine vaccination including a catch-up campaign (interventions II-V) would still be the preferred strategy in all sites, depending on the WTP threshold (S10 Figure). If two doses are required for children <5 years of age, the optimal strategy would be no vaccination up to a WTP threshold (which varied by setting and vaccine cost) above which interventions IV or V would be optimal in all settings but Lwak; less ambitious strategies would not confer sufficient benefits to justify the costs of administering two doses (S11 Figure). Routine vaccination alone (intervention I) was unlikely to be cost-effective in Lwak under a two-dose schedule (S9 Figure).

### 3.4 Sensitivity analysis

**Fig. 4:**
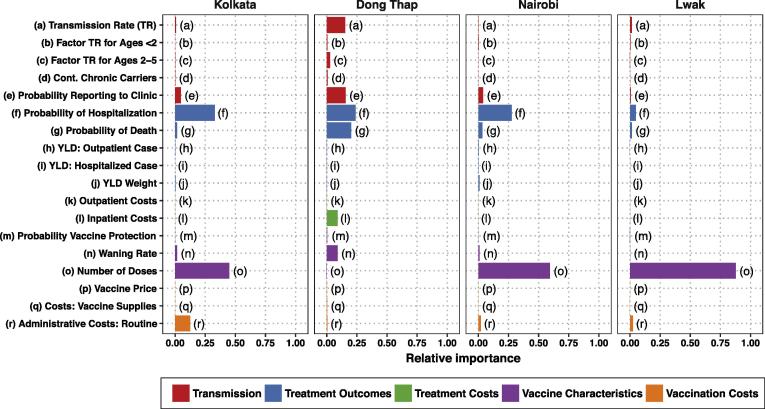
Impact of uncertainty in each parameter on the net monetary benefits of routine vaccination as compared to the status quo, estimated using random forest analysis.

In all settings but Dong Thap, the number of doses contributed most to uncertainty in the NMB (as evaluated at a WTP equal to one GDP per capita) for routine vaccination, followed by the hospitalization rate (Fig. 4). In Dong Thap, the hospitalization rate and the probability of death were the most influential parameters. When we considered the NMB of delivery strategies II–V (S12 Figure), the most influential parameters did not change remarkably.

Interestingly, some of the vaccine-related parameters about which we had the least amount of data (vaccine price and probability of protection) did not rank highly in importance. However, the operational costs of vaccination (for which we applied a wide variance in the absence of reported data) figured prominently in Kolkata and Lwak. We omitted Delhi from sensitivity analyses because parameter uncertainty had no bearing on the conclusions regarding the optimal strategy.

## 4 Discussion

Our analysis is the first to evaluate the cost-effectiveness of TCV delivery strategies. Most strikingly, we found that although routine infant vaccination alone (intervention I) was likely to be cost-effective (or even cost-saving) compared to the status quo, it was not the optimal intervention when compared to strategies that include one-time catch-up campaigns (interventions II–V). In Delhi and Dong Thap, the most ambitious intervention (V) was cost-saving or optimal at WTP levels well below the WHO threshold for very cost-effective interventions, even when accounting for sizeable uncertainty in model parameters. In Kolkata and Nairobi, where incidence is moderate, no strategy was consistently predicted to be superior, but routine vaccination alone (intervention I) was consistently dominated by more ambitious strategies.

Previous cost-effectiveness analyses have recommended the introduction of ViPS vaccines in Delhi and Kolkata [38], [39], but since ViPS is not licensed for children <2 years of age, those analyses did not consider infant immunization. In addition, previous analyses were based on static disease models rather than dynamic transmission models, which overlook the potential herd immunity benefits of vaccinating older age groups who are less susceptible to clinical disease but may contribute to typhoid transmission. Herd immunity accounted for 28–43% of the cases averted in our model, depending on the setting and delivery strategy (S13 Figure), and had a substantial impact in our findings. In general, vaccination was not as likely to be cost-effective and less ambitious strategies were preferred when we did not account for herd immunity (S9 Table).

There is considerable uncertainty in the burden of typhoid fever, severity (as indicated by the probability of hospitalization in this analysis), costs associated with treatment, and vaccine costs and effectiveness. We conducted sensitivity analyses to identify the most influential parameters within each setting while accounting for high-level interactions. Some of the parameters for which we had the least amount of data to inform (e.g. treatment costs in Kenya and Dong Thap) did not have a strong influence on the NMB or the conclusions drawn from our model, but more precise estimates of the probability of hospitalization in Kolkata, Nairobi, and Lwak could yield stronger evidence for one intervention over others (S12 Figure). This approach, coupled with value of information analyses, could inform future research priorities.

Based on our scenario analysis, a one- vs two-dose schedule could have a formidable impact on the decision to introduce TCV; for instance, no strategy was cost-effective in Lwak when a two-dose schedule was required. When we examined the interaction between dosing schedules and vaccine price, strategies implementing two-dose schedules were likely to be cost-effective in Kolkata and Nairobi if the vaccine price was ≤I$2/dose (S11 Figure). Of the vaccines currently in development or production, only Typbar-TCV requires one dose; PedaTyph^TM^ (BioMed) requires two doses for children <2 years old and others require two doses for children <5 years old [15].

We also evaluated a range of vaccine prices in scenario analyses, based on the prices of other Gavi-supported vaccines [40]. While the vaccine price had some impact on the preferred delivery strategy, routine vaccination with TCV was still likely to be cost-effective in all settings except Lwak when the cost per dose was I$5 assuming a one-dose schedule (S9 Figure). However, at the current price of TCV-Typbar on the private market in India (1800 rupees = I$106), no strategy is cost-effective in Kolkata, Nairobi, or Lwak; once again, strategies that coupled one-time campaigns with routine vaccination were optimal and likely to be cost-effective in Delhi and Dong Thap, where the costs of treatment were high (S14 Figure).

In resource-constrained settings, the decision to adopt an intervention is made not only with an eye towards cost-effectiveness, but also affordability [41]. Therefore, throughout our analysis, we made a number of conservative choices to bias against vaccine adoption, thereby lowering the risk of displacing existing or planned interventions that may confer a higher benefit to the population. For example, we assumed deaths only occurred among hospitalized cases. More broadly, we avoided overstating the case for TCVs by adopting the healthcare payer’s perspective, which disregards numerous sources of the economic burden such as caregiver’s time, transportation costs to the clinic, and foregone wages [42]. Despite these conservative choices, we found that most TCV delivery strategies were cost-effective at low WTP thresholds, and in some settings these strategies were even cost-saving.

There are three factors that we have not taken into account that may raise the willingness-to-pay of these interventions. First, three of the incidence studies were carried out in urban “slums” (in Delhi, Kolkata, and Nairobi), which may represent a higher incidence of typhoid than in the rest of the city; therefore, we recommend that all policy decisions be carried out taking into consideration the possible heterogeneity of incidence in a setting. Second, the incidence of typhoid varies over time, and has decreased in some settings in the time since the studies took place; in some instances, this may be due to improvements in water and sanitation infrastructure [43], [44], [45]. However, while improvements in clean water and sanitation often lead to decreases in typhoid transmission, the relationship is not well quantified and we do not have information on plans regarding the implementation of broad infrastructure projects and their expected impact (which would likely be small over the 10-year time horizon of our primary analysis) [43], [44], [46]. If the transmission rate of typhoid were to decrease over time in the comparator (no vaccination) scenario, this would raise the willingness-to-pay for any particular vaccination strategy, but the overall conclusions would be similar (S15 Figure). Third, evidence to date suggests that there are no serious adverse events caused by any of the modern typhoid vaccines [15], [47]. If, however, evidence emerges as to the nature and rate of occurrence of such events, they could be incorporated as additional “costs” of vaccination, thereby increasing the cost per DALY averted for any given vaccination strategy.

In order to generalize the findings of our analysis to all LMICs where typhoid remains endemic, additional work is needed to better understand how variation in the epidemiology and costs of typhoid fever between locations (which far outpaces the variance within locations) could result in different recommendations. Furthermore, post-introduction surveillance activities would help to validate and refine model predictions of the potential long-term effects of vaccination on disease dynamics, which in turn could help policy-makers in other locations. However, the current analysis demonstrates that TCVs are an economically viable tool to control the burden of typhoid fever in low-resource settings. Decisions regarding the recommended use of TCVs, including an updated WHO position paper and Gavi vaccine investment strategy, are imminent and must not be held hostage to the need for more data [16]. Robust analyses such as this allow for the use uncertain or imperfect data to judiciously inform programmatic and research priorities.

## Declaration of interests

The authors declare no conflicts of interest exist.

## Author contributions

MA, JB, VEP conceived the study and wrote the initial draft; MA, JB performed the analyses; ADP, VEP helped with the analyses; all authors edited and approved the final draft.

## Funding

Bill and Melinda Gates Foundation and Research Foundation – Flanders (FWO).

## Appendix A Supplementary material

Supplementary data 1 Supplemental methods and results.
