# Supplementary material for: Cost-effectiveness analysis of typhoid conjugate vaccines in five endemic low- and middle-income settings
Source: Vaccine. 2017 Jun 14;35(27):3506–14. doi: 10.1016/j.vaccine.2017.05.001 (PMC5462484; doi:10.1016/j.vaccine.2017.05.001)
Supplement: Supplementary data 1 — Supplemental methods and results. [file mmc1.docx]

**Supplementary Text**

***Cost-effectiveness analysis of typhoid conjugate vaccines in five endemic low- and middle-income settings***

Marina Antillón, M.Phil.^1*^, Joke Bilcke, Ph.D.^1,2^, A. David Paltiel, Ph.D.^3^, Virginia E. Pitzer, Sc.D.^1^

*^1^Department of Epidemiology of Microbial Diseases, Yale School of Public Health, New Haven, CT 06520-8034 USA*

^2^ *Center for Health Economics Research and Modeling Infectious Diseases, University of Antwerp, Belgium*

*^3^ Department of Health Policy and Management, Yale School of Public Health, New Haven, CT 06520-8034 USA*

**Table of Contents**

**1** **Dynamic transmission model** 2

1.1 Model structure 2

1.2 Estimating transmission parameters 4

**2** **Adjustments to the observation process** 5

**3** **Estimating the contribution of chronic carriers to transmission** 8

**4** **Conjugate vaccine protection** 9

**5** **Estimating disability adjusted life-years** 10

**6** **Treatment and economic parameters** 11

**7** **Sensitivity Analyses** 19

**8** **Additional References** 22

**9** **Appendix Tables** 23

**10** **Appendix Figures** 32

# **Dynamic transmission model**

We modified a previously developed compartmental model for the transmission dynamics of typhoid fever,[1] depicted in S1 Figure The compartmental model is specified as a system of ordinary differential equations (ODEs):

| Without vaccination | With vaccination |
| --- | --- |
| $\frac{dS}{dt}=bN-\lambda S$  $\frac{dI_{M}}{dt}=\lambda S-\delta I_{M}$  $\frac{dR}{dt}=\delta(1-\theta-\alpha)(I_{M}+I_{Q})-\omega R$  $\frac{dC}{dt}=\delta\theta(I_{M}+I_{Q})$  $\frac{dS_{R}}{dt}=\omega R-\lambda S_{R}$  $\frac{dI_{Q}}{dt}=\lambda S_{R}-\delta I_{Q}$ | $\frac{dS}{dt}=bN-\lambda S-\kappa\nu S+\omega V_{1}$  $\frac{dI_{M}}{dt}=\lambda S-\delta I_{M}$  $\frac{dR}{dt}=\delta\left( 1-\theta-\alpha\right)\left( I_{M}+I_{Q} \right)-\omega R-\kappa\nu R$  $\frac{dC}{dt}=\delta\theta(I_{M}+I_{Q})$  $\frac{dS_{R}}{dt}=\omega R-\lambda S_{R}-\kappa\nu S_{R}$  $\frac{dI_{Q}}{dt}=\lambda S_{R}-\delta I_{Q}$  $\frac{dV_{1}}{dt}=\kappa\nu S- \omega_{V}V_{1}$  $\frac{dV_{2}}{dt}=\kappa\nu(S_{R}+R)- \omega_{V}V_{2}$ |

where *N* is the total population size (i.e. sum of the model compartments, as defined in Figure A1 and below). The model parameters are defined in S2 Table. In order to estimate the transmission parameters and the reporting rate, we fit the transmission model (without the vaccination compartments) to observed incidence data. We then simulated the impact of vaccination using the full transmission model.

## Model structure

The model assumes an individual is born into the completely susceptible compartment (***S***) of the lowest age-stratum. In the absence of vaccination, the individual contracts infection with typhoid at a rate ***λ*** (also known as the force of infection) and moves on to compartment ***I_M_*** (infectious, with a possibility of symptoms). Some fraction of symptomatic and infectious individuals will present to a healthcare provider and a fraction of those will warrant hospitalization. A small proportion of hospitalized cases will be fatal. Following primary infection, a small fraction of infectious individuals will become life-long chronic carriers, designated to compartment ***C***. The remaining cases will recover, temporarily gaining immunity to reinfection and moving on to compartment ***R***. Following the waning of temporary immunity, individuals enter compartment ***S_R_***, which represents individuals who have been previously infected and are susceptible to reinfection. However, we assume subsequent infections, which again occur at a rate ***λ*,** are subclincal (***I_Q_***); this assumption is necessary in order to reproduce the age distribution of typhoid cases in endemic settings [2–4]; previous efforts to estimate the rate of waning immunity to clinical infection resulted in estimates that were not significantly different from zero [5]. Again, infectious individuals are assumed to recover into the ***R*** compartment and be temporarily immune to reinfection upon recovery. We assumed that subclinical cases (***I_Q_***) do not go on to become chronic carriers for two reasons: 1) data to parameterize the probability of becoming a chronic carrier is only available from studies which followed symptomatic typhoid cases,[6] and 2) assuming that subclinical cases become chronic carriers at the same rate resulted in populations with upwards of 50% chronic carriers in the oldest age group, which is not supported by surveys of chronic carriers in Asia and Latin America.[7,8]

Symptomatic and subclinical infectious individuals (***I_M_*** and ***I_Q_***) and chronic carriers (***C***) contribute to the force of infection. Chronic carriers are assumed to shed the pathogen continuously at a fraction ***r*** of the rate of short-term infectious individuals over the rest of their life. The force of infection (***λ_a_***) depends on age (*a*) and is the product of the transmission rate, ***β_a_***, and a weighted sum of individuals in compartments ***I_M_, I_Q_,*** and ***C*** divided by the total population size ***N***:

$$\boldsymbol{\lambda}_{\boldsymbol{a}}\boldsymbol{=}\frac{\boldsymbol{\beta}_{\boldsymbol{a}}}{\boldsymbol{N}}\sum_{\text{All ages}} \boldsymbol{(I}_{\boldsymbol{M}}\boldsymbol{+}\boldsymbol{I}_{\boldsymbol{Q}}\boldsymbol{+rC)}$$

The best fit models were those that allowed for a different transmission rate for children in the two youngest age groups: children 0-2 years of age and children 2-5 years of age. This is consistent with the studies in our analyses as well as others.[9–11]

Unlike previous versions of our model, we do not distinguish between short- versus long-cycle transmission (the latter occurring at a slight delay). These two transmission routes are not individually identifiable from the aggregate annual incidence rate data, which we used for model fitting. For simplicity, we assumed all transmission occurs at a rate that is directly proportional to the number of currently infectious individuals. This assumption should not affect our conclusions regarding the impact of vaccination.

To simulate vaccination, two additional compartments were added to the model. One compartment, ***V_1_*** denotes persons who were immunologically naïve to typhoid fever (i.e. never infected) and successfully mounted a protective immunological response to vaccination. A second compartment, ***V_2_***, denotes successfully immunized individuals who had been previously infected, but whose natural immunity has partially waned. These individuals are protected from subclinical infection, but were already partially immune to clinical infection.

## Estimating transmission parameters

We employed a Hamiltonian Monte Carlo (HMC) algorithm to sample from the joint posterior distribution of unknown model parameters from each of the sites in our analysis [12]. The joint posterior distribution is equal to the product of the prior distributions of the unknown parameters and the Poisson likelihood of the observed incidence. The Poisson likelihood is parameterized with rate parameter equal to the simulated age-specific incidence and adjusted for three binomial sub-processes of the observation process. Since the convolution of a Poisson process and a binomial process is a Poisson process with rate parameter equal to the product of the rate and probability parameters, our likelihood function is structured as follows:

$$\text{Observed Cases}\boldsymbol{|}\text{Population}\boldsymbol{\sim}\text{Poisson}\boldsymbol{(}\boldsymbol{\Lambda}_{\boldsymbol{a,j}}\boldsymbol{\phi}_{\boldsymbol{j,a}}\boldsymbol{\phi}_{\boldsymbol{c,j,a}}\boldsymbol{\phi}_{\boldsymbol{r,j}}\boldsymbol{)}$$

where$\boldsymbol{\Lambda}_{\boldsymbol{a,j}}$ is the simulated yearly incidence at endemic equilibrium in age group ***a*** and site ***j***, $\boldsymbol{\phi}_{\boldsymbol{p,j,a}}$ is the participation probability, $\boldsymbol{\phi}_{\boldsymbol{c,j,a}}$ is the diagnostic accuracy of blood culture for the corresponding age group ***a*** in site ***j***, and $\boldsymbol{\phi}_{\boldsymbol{r,j}}$is the probability of having symptoms and reporting to the study clinic in site ***j***. The participation rate (defined as the probability the individual consented to the study and had a blood sample drawn) was reported in each of the studies, and the diagnostic accuracy of blood culture was given a prior distribution as described in Section 2 of this supplement. We set uninformative priors for the parameters that described the transmission rate and the reporting probability, therefore allowing the incidence data to determine the posterior distribution of these parameters.

To simulate the yearly incidence of typhoid at endemic equilibrium for each age group, $\boldsymbol{\Lambda}_{\boldsymbol{a,j}}$, we assumed a constant population size and no change in the age structure of the population. The simulated yearly incidence was the cumulative sum (over one year) of all transfers from the ***S*** compartment to the ***I_M_*** compartment in the ODE model. We initialized the model with one infectious individual and a completely susceptible population. We integrated the ODE system for 100 years prior to evaluating the model-predicted incidence, by which time we determined (by visual inspection) that it had reached endemic equilibrium.

We computed the posterior distributions of transmission model parameters using Stan, an open-source probabilistic programming language.[12] We drew 5,000 samples from the joint posterior distribution of model parameters for each site. The potential scale reduction factor, $\hat{R}$, approximated 1.0 for all chains, confirming that the Monte Carlo chains had converged.[13] Since the smallest effective sample size of our parameters was >1,000, we applied a thinning parameter of 5 to eliminate auto-correlation among samples. Summaries of the marginal posterior distributions are shown in S2 Table.

# **Adjustments to the observation process**

The typhoid surveillance studies featured in our analysis did not follow a single, unified protocol, and therefore, the observation process among the sites had to be taken into account.[14–17] Besides the estimated probability of reporting to the study clinic, $\boldsymbol{\phi}_{\boldsymbol{r,j}}\boldsymbol{,}$ we took into account two additional sources of heterogeneity in the observation process that produced the data: 1) the probability that an eligible, care-seeking case enters the study and a blood sample is taken, and 2) the sensitivity of blood culture as a function of sample volume. The parameters related to these processes were informed by data in the studies and in the broader scientific literature, as described below.

First, the probability that an eligible case enters the study is a result of operational constraints and willingness to participate. In Kenya, for example, it was not feasible to culture the blood for all eligible patients that presented with prolonged fever to study sites because of the high incidence of fever cause by malaria [17]. Therefore, we modeled the probability that a true typhoid case would participate in the study (i.e. attend a study hospital and have blood drawn for diagnosis) as a binomial process:

$$\text{Cases that agreed to participate}\boldsymbol{\sim}\text{Binomial}\boldsymbol{(}\text{Cases that reported to study site}\boldsymbol{,}\boldsymbol{\phi}_{\boldsymbol{p,j,a}}\boldsymbol{)}$$

The “participation rate” $\boldsymbol{\phi}_{\boldsymbol{p,j,a}}$ was set equal to the reported (age-specific) participation rate in study site *j*, which was reported in all studies (Tables S3-S7).

We must make a special note with regards to the incidence in Kenya. The adjustment we have detailed above takes into account the study authors’ “extrapolation 1” but not their “extrapolation 2”, which accounts for individuals who reported attending a clinic other than the study clinic for febrile illness. We refrained from applying the second extrapolation because we did not have analogous data from the other studies regarding attendance to other clinics and we did not want to treat the data differently across sites. Cases that have reported to other clinics influence the reporting parameter $\boldsymbol{\phi}_{\boldsymbol{r,j}}$**,** which is estimated using the data in all sites.

Second, the probability that an eligible case is reflected in our data depends on whether the blood culture of a patient who has entered the study and is infected typhoid results in a positive diagnosis. The test sensitivity of blood culture has been estimated to be between 40% and 60%; therefore, adjusting for blood culture sensitivity is important in order to avoid underestimating the true burden of the disease.[18–23] It is widely recognized that sensitivity is correlated with the volume of blood taken from a patient, but this correlation has not been quantified. Since blood samples collected from younger children are typically smaller than the samples collected from older children and adults, it is imperative to understand the relationship between sample volume and blood culture sensitivity in order to correctly estimate age-related differences in incidence.

We assumed the number of observed (culture-confirmed) typhoid cases in age group *a* in each surveillance site *j* represented a fraction $\boldsymbol{\phi}_{\boldsymbol{c,j,a}}$of the estimated number of cases that would have been detected using a perfect (100% sensitive and specific) test:

$$\text{Observed blood-culture positive cases}\boldsymbol{\sim}\text{Binomial}\left( \text{Cases that agreed to participate}\boldsymbol{,}\boldsymbol{\phi}_{\boldsymbol{c,j,a}} \right)$$

We surveyed the diagnostic accuracy literature on blood culture sensitivity of typhoid cases. We only considered studies in which cases were confirmed by bone marrow culture (the gold standard), which is estimated to have a sensitivity of 90-100%.[18–23] A meta-regression allowed us to estimate the correlation between the amount of blood drawn and culture sensitivity, which we used to parameterize the prior distribution for $\boldsymbol{\phi}_{\boldsymbol{c,j,a}}$ above.[24]

One set of studies measured the test sensitivity of patients using two different amounts of blood from the same patients, which allowed us to understand whether an additional mL of blood would render the blood culture of a typhoid case positive.[20,23,25] To accommodate these studies, we chose a probability function analogous to a survival function. The probability that a blood culture was positive at volume $\nu_{1}$ is denoted as:

$$\boldsymbol{\pi}_{\boldsymbol{1}}\boldsymbol{=}\mathbf{1-}\exp\left( \mathbf{-}\boldsymbol{\psi}\text{ × volume}_{\boldsymbol{1}} \right)$$

The probability that a blood culture was negative at volume $\nu_{1},$ but positive at volume $\nu_{2}$ is:

$$\boldsymbol{\pi}_{\boldsymbol{2}}\mathbf{=[1- exp}\left( \mathbf{-}\boldsymbol{\psi}\text{ × volume}_{\boldsymbol{2}} \right)\mathbf{]- [1-exp}\left( \mathbf{-}\boldsymbol{\psi}\text{ × volume}_{\boldsymbol{1}} \right)\mathbf{]}$$

Lastly, the probability that a (bone-marrow-confirmed) typhoid patient was blood-culture-negative using the largest sample taken is:

$$\boldsymbol{\pi}_{\boldsymbol{3}}\boldsymbol{=}\mathbf{exp(-}\boldsymbol{\psi}\text{ × volume}_{\boldsymbol{2}}\mathbf{)}$$

In other words, patients whose blood culture was negative at volume $\nu_{2}$ were treated as right-censored observations—more than the amount of blood collected would be necessary for the blood culture to be positive. To draw from the posterior distribution of $\boldsymbol{\psi}$, the above vector of probabilities were used to calculate the multinomial likelihood of the observed number of cases who tested positive at volume *v* in each study:

$$\boldsymbol{Observation \sim Multinomial(\pi, All typhoid cases in the study)}$$

Some studies estimated the test sensitivity of patients using a fixed volume of blood; that is, some studies did not compare sensitivity between samples of different volumes.[18,19,21,22] These studies had a likelihood function that reduced to a binomial distribution. The overall posterior distribution had a density equal to the product of the likelihood function for each study.

Lastly, because unmeasured heterogeneity between studies may impact the observations, we specified the $\boldsymbol{\psi}$ parameter as a random effect with a prior log-normal distribution with two hyper-parameters.

$$\boldsymbol{\psi}_{\boldsymbol{j}}\boldsymbol{\sim}\mathbf{Log}\text{Normal}\left( \boldsymbol{\mu, \sigma} \right)$$

The hyperparameters and the random effects were sampled jointly using JAGS software. The observed sensitivity estimates and our model estimates are shown in S2 Figure.

Posterior predictions of sensitivity were made by simulating $\boldsymbol{\phi}_{\boldsymbol{c,j,a}}$ as a function of the random effect hyper-parameter, $\boldsymbol{\mu}$.

$$\boldsymbol{\phi}_{\boldsymbol{c,j,a}}\boldsymbol{=}\mathbf{1-}\exp\left( \boldsymbol{-\mu}\text{ × volume}_{\boldsymbol{1}} \right)$$

for the blood sample volumes listed in Tables S3-S7. We chose not to make any inference on the sensitivity of blood culture for samples smaller than 2 mL since no incidence study collected fewer than 2 mL of blood.

# **Estimating the contribution of chronic carriers to transmission**

We estimated a prior distribution for the relative infectiousness of chronic carriers (***r***) by leveraging data from a cluster-randomized study of Vi-polysaccharide vaccine in Kolkata. As previously shown, the indirect protection conferred on a population is a function of the contribution of chronic carriers to the overall force of infection.[1] The contribution of the chronic carriers is the product of two components: 1) the prevalence of chronic carriers in the population and 2) the relative infectiousness of chronic carriers (***r***) as compared to acute typhoid infections. The prevalence of chronic carriers (the first component) was based on the model output, while the trial data allowed us to estimate the relative infectiousness of chronic carriers (the second component).

We fit the transmission model to the incidence data in Kolkata while fixing ***r*** at values from 0.01 to 1 in increments of 0.01. We estimated the transmission rate parameter conditional on each value of ***r***, and then simulated the overall and indirect effects of a mass vaccination campaign using the Vi-polysaccharide vaccine, assuming 60% coverage of individuals 2 years of age and older, as described in Sur et al.[26] Briefly, the indirect effect of vaccination is the percent decrease in incidence among unvaccinated individuals in a partially vaccinated population compared to the incidence in a completely unvaccinated population. The overall effect is defined as the incidence among both vaccinated and unvaccinated individuals in the population with vaccination compared to the incidence in the unvaccinated population.[27] We then evaluated the likelihood of each parameter set (including ***r***) by comparing the value of the simulated indirect effect against the observed indirect effect in the trial, which we parameterized using a beta distribution according to the method of moments (*IE*~Beta(3.27,4.16)). We recovered a comparable sample of parameter sets if we evaluated the likelihood against the observed overall effect (*OE*~Beta(18.00,13.58)).

In summary, for each of the 100 values of r from 0.01 to 1 we:

1) Estimated the transmission parameter (***β***) conditional on the fixed value of ***r***.

2) Simulated the impact of vaccination and calculated the model-predicted indirect effect.

3) Evaluated the likelihood of the model-predicted indirect effect based on the current parameter set (***r***, ***β***) given the beta-distributed observed indirect effect.

We then sampled from the 100 values of ***r*** weighted by the likelihood in step 3. Finally, we fit a beta distribution to the sampled values of ***r***, resulting in Beta(3.69, 6.79), which we used as the prior distribution for ***r*** when we fit the dynamic model to the incidence data in all sites.

# **Conjugate vaccine protection**

To estimate the probability of protection and the rate of waning immunity of TCV, we used data from a study that measured vaccine efficacy for the Vi-rEPA conjugate vaccine three times over the period of 46 months, shown in S8 Table.[28,29] Although Vi-rEPA is no longer the leading TCV candidate, it is the only vaccine for which clinical endpoints have been documented over a sufficiently long time to estimate duration of protection. Clinical endpoints have not been evaluated for other TCVs except PedaTyph, for which recipients have not been followed up for a long enough period to estimate the rate of waning immunity.[30] Several other candidates have been proven safe and are believed to be protective based on serological surrogates of immunity.[31–33] We modeled the vaccination and infection process using a simple simulation, as depicted in S3 Figure.

The incidence rate (***γ***) was assumed to be constant over time, and was estimated by sampling from a gamma distribution with shape parameter equal to the number of infections observed in the unvaccinated cohort over each period of follow-up and the scale parameter equal to the person-time of observation among unvaccinated subjects over that period.

The state of the vaccinated cohort over time was described according to the following system of ODEs:

$$\frac{\boldsymbol{d}\boldsymbol{V}_{\boldsymbol{P}}}{\boldsymbol{dt}}\boldsymbol{= -}\boldsymbol{\omega}_{\boldsymbol{V}}\boldsymbol{V}_{\boldsymbol{p}}$$

$$\frac{\boldsymbol{d}\boldsymbol{S}_{\boldsymbol{V}}}{\boldsymbol{dt}}\boldsymbol{=}\boldsymbol{\omega}_{\boldsymbol{V}}\boldsymbol{V-}\boldsymbol{\gamma S}_{\boldsymbol{v}}$$

$$\frac{\boldsymbol{d}\boldsymbol{I}_{\boldsymbol{V}}}{\boldsymbol{dt}}\boldsymbol{=\gamma}\boldsymbol{S}_{\boldsymbol{v}}$$

where ***ω_V_*** is the rate of waning immunity and ***γ*** is the incidence rate The integral for the above ODE is analytically tractable, and assuming that the initial values are ***V_P_= νV, S_V_=(1-ν)V***, and ***I_V_=0***, then the number of individuals in each compartment at time ***t*** are defined by the following equations:

$$\boldsymbol{V}_{\boldsymbol{P}}\left( \boldsymbol{t} \right)\boldsymbol{=\nu V}\text{exp}\boldsymbol{(-}\boldsymbol{\omega}_{\boldsymbol{V}}\boldsymbol{t)}$$

$$\boldsymbol{S}_{\boldsymbol{V}}\left( \boldsymbol{t} \right)\boldsymbol{=}\frac{\boldsymbol{\omega}_{\boldsymbol{V}}\boldsymbol{\nu V}\left( \text{exp}\left( \boldsymbol{-\gamma t} \right)\boldsymbol{-}\exp\left( \boldsymbol{-}\boldsymbol{\omega}_{\boldsymbol{V}}\boldsymbol{t} \right) \right)}{\boldsymbol{\omega-\gamma}}\boldsymbol{+}\left( \boldsymbol{1-\nu} \right)\boldsymbol{V}\exp\left( \boldsymbol{-\gamma t} \right)$$

$$\boldsymbol{I}_{\boldsymbol{V}}\left( \boldsymbol{t} \right)\boldsymbol{=\nu V}\left( \boldsymbol{1-}\frac{\boldsymbol{\gamma}\exp\left( \boldsymbol{-}\boldsymbol{\omega}_{\boldsymbol{V}}\boldsymbol{t} \right)\boldsymbol{-}\boldsymbol{\omega}_{\boldsymbol{V}}\mathbf{exp}\boldsymbol{(-\gamma t)}}{\boldsymbol{\gamma-}\boldsymbol{\omega}_{\boldsymbol{V}}} \right)\boldsymbol{+}\left( \boldsymbol{1-\nu} \right)\boldsymbol{V}\mathbf{(1-exp} \left( \boldsymbol{-\gamma t} \right)\boldsymbol{)}$$

The infected compartment in this model represents the cumulative infection rate from time 0 until time ***t***. The infected individuals over ***t_i-1_*** and ***t_i_*** are equal to ***I_V_(t_i_) – I_V_(t_i-1_).*** The posterior distribution of the rate of waning immunity (***ω***) and the probability of protection immediately following vaccination (***ν***) were then sampled via a Metropolis-Hastings algorithm using the JAGS software.[34] We assumed the observed number of cases in the vaccinated cohort over each follow-up period was Poisson distributed with rate parameter equal to the model-predicted incidence of infection throughout the time of follow-up. We applied non-informative prior distributions to the rate of waning immunity and vaccine protection parameters. The posterior distributions for the rate of waning vaccine-induced immunity and the probability of protection are highly correlated; therefore, we sampled directly from the joint distribution in order to simulate the impact of vaccine interventions.

# **Estimating disability adjusted life-years**

As per recommendations of the Bill and Melinda Gates Foundation’s reference case and World Health Organization guidelines for the conduct of cost-effectiveness analyses, we evaluated the effectiveness of each TCV delivery strategy in terms of disability-adjusted life-years (DALYs). Costs and effects were discounted at a rate of 3% per year.[35,36] DALYs are defined as the sum of the years of life lost to disability (illness) (YLD) and the years of life lost by fatal cases (YLL).[35,37] A disability weight, a factor between 0 (perfect health) and 1 (equivalent to death), was applied to years of life lost to illness (but not to death) to reflect the severity of typhoid relative to other diseases. We used the disability weights for typhoid fever from the 2010 Global Burden of Disease Studies (see section 6.3 of this supplement).[38–41]

We assumed that inpatient cases accrued more DALYs than outpatient cases because the severity of typhoid is worse for inpatient cases, which is correlated with the time to recovery. Moreover, we assumed all outpatient cases recovered and that deaths only occurred among inpatient cases for two reasons. First, hospitalization is correlated with disease severity; we assumed that cases so severe as to result in death would warrant hospitalization.[2,14] Second, restricting fatalities to the inpatient population would be, at worst, a conservative bias that would prevent us from overstating the case for vaccine introduction.

Because our analysis is from the healthcare payer’s perspective, final DALY estimates included typhoid cases that reported to care, including eligible individuals who were not tested or who (falsely) tested negative for typhoid. Culture-negative cases were assumed to follow the same distributions for the costs of care and the probability of hospitalization and death as culture-positive cases.

# **Treatment and economic parameters**

We used published estimates to parameterize treatment outcomes and economic parameters. The treatment parameters were the duration of disease, the probability of hospitalization, and the probability of death among hospitalized patients. The cost parameters included the cost of inpatient and outpatient treatment and vaccination costs (price, operational costs, supplies). We parameterized the uncertainty distributions using the published standard error and the method of moments. All probabilities or ratios were parameterized with Beta distributions and all costs were parameterized with Gamma distributions unless otherwise noted. Our general rule was the less information we had, the wider the uncertainty distribution, and when no standard error (or standard deviation) was reported, we assumed that the uncertainty interval of the estimate was bounded by values 75% above and below the estimate. Therefore, we calculated the standard error using the following equation:

$$\text{SE}\boldsymbol{=}\text{Mean}\boldsymbol{\times}\frac{\boldsymbol{0.75}}{\boldsymbol{1.96}}$$

All of the data we used to parameterize the costs of treatment and vaccination came from micro-costing studies, except for vaccine administrative costs in India.[42–44] No treatment cost data were available for Kenya, so we used data from a typhoid costing study performed in Tanzania, and validated those costs against the costs of treatment for malaria in Kenya (see below).[45,46] No treatment cost data were available for Dong Thap, so we leverage data from a costing study performed in Hue, Vietnam.[47] We have adjusted for inflation and converted costs to international dollars using the following formula:

$$\frac{\text{Consumer price index in 2015}}{\text{Consumer price index in year of estimate}}\times\frac{\text{Official exchange rate (LCU per US\$) in year of estimate}}{\text{PPP conversion factor, GDP (LCU per international \$) in year of estimate}}$$

These conversion factors were retrieved from the World Bank’s World Development Index database [48].

Survey data on vaccine administration costs were available in Kenya and Vietnam, but not in India; we used estimates reported in the India country Multi-Year Plan (cMYP).[42–44] In Vietnam, we assumed that typhoid vaccine administration costs were equal to HPV vaccine administration; however, HPV vaccine administration costs often include the cost of a short counseling session, potentially biasing our analysis against vaccine introduction. The data available allowed us to parameterize costs specific to routine and campaign vaccination in India and Vietnam, but not in Kenya.[42–44] Because we did not have data itemized by each of the components of operational vaccination costs, we assumed full operational costs (rather than incremental costs); at worst, this assumption lends our analysis a conservative bias against vaccine adoption.

1. *Probability of hospitalization*

Each of the incidence studies reported the proportion of patients that were hospitalized. In Kolkata, 2 out of 94 patients were hospitalized; in Delhi, 6 out of 63 patients were hospitalized; in Nairobi, 2 out of 135 patients were hospitalized; and in Lwak, 6 out of 22 patients were hospitalized. No data regarding the probability of hospitalization was available in the Dong Thap surveillance study, but a subsequent vaccine study in Dong Thap reported that 21 out of 61 children were hospitalized.[14–17]

Because of the heterogeneity in the probability of hospitalization, we applied a shrinkage estimator to provide more conservative estimates of hospitalization. Shrinkage estimators tend to bias parameter estimates towards a global mean, therefore minimizing the impact of site-specific anomalies in the data. We drew 10,000 samples of the estimator for each site using JAGS software, and then selected 1,000 random samples to use in our cost-effectiveness analysis.

1. *Expected duration of typhoid illness (years of life lost to disability – YLD) for inpatients and outpatients*

The uncertainty around the average duration of typhoid illness for an inpatient was characterized by a uniform distribution, varying between 2 and 4 weeks. The smallest estimate of duration reported in the literature was 10.2 days after treatment began, but no typhoid cases were considered that had not experienced 3 days of fever. The highest estimate reported in the literature was 26 days for children in a study in Tanzania, who bear the majority of the burden; once again, we assumed that 26 days was an estimate of the duration after care was sought.[46]

We estimated the YLD for outpatient cases as a proportion of the YLD for inpatient cases to ensure that our model always assigned a longer YLD for more serious cases that require hospitalization. We bounded the uniform distribution for the factor for outpatient YLD at 0.25-0.75. This results in a lower bound of YLD of 3.5 days (14*0.25), consistent with the fact that most studies only count typhoid patients who have experienced fever for more than 3 days, and 21 days, consistent with the highest duration of disease reported in the Diseases of the Most Impoverished (DOMI) studies.[47]

1. *Disability weight for typhoid*

The Global Burden of Disease Study (GBD) in 2010 and 2013 classified the “health states” (symptoms) of typhoid fever as comparable to moderate or severe acute infections, and they carried out surveys to calculate disability weights (DW).[40,41] Typhoid fever cases with intestinal perforation were considered in a separate category, and to be conservative (bias against vaccination) we have chosen not to consider the proportion of those cases in the population in our analysis. We decided to use the DWs for GBD 2010 in our analysis because the 2013 DWs were calculated using data for which more than half the sample was from developed countries.[38,41] In GBD 2010, moderate infections had a DW of 0.053 (0.033-0.081) and severe infections had a DW of 0.210 (0.139-0.298).[38] Since it is unclear how many infections fall in the category of “moderate” or “severe” in any given population, we have chosen to incorporate this uncertainty as follows:

**Disability weight for moderate infection ~ Normal (0.053, 0.01)**

**Disability weight for severe infection ~ Normal (0.21, 0.04)**

**Probability of severe case ~ Uniform(0,1)**

**Disability weight ~ DW(moderate)(1-Pr(severe)) + DW(severe)*Pr(severe)**

Therefore, our final distribution for the disability weight is normally distributed with a mean of 0.13 and a 95% confidence interval of (0.03, 0.23).

1. *Probability of death*

We parameterized the probability of death (conditional on hospitalization) using mortality data from one study that estimated the fatality rate among drug-sensitive and drug-resistant cases.[49] Because that study did not find a statistically significant difference in mortality between drug-sensitive and drug-resistant cases, we used the pooled estimate of mortality for our distribution, modeling the probability of death as Beta(19, 1139). We reviewed other mortality studies among hospitalized patients and found this distribution was consistent with those studies. We excluded studies among patients with intestinal perforation or very severe cases, which would bias our estimate of mortality upwards.[50,51]

1. *Years of life lost (YLL) for fatal outpatient cases*

To estimate the YLL, we took the product of the simulated number of deaths in each age-stratum and the years of life that a person in that stratum would have lived. We assumed that the average person in any stratum would live a quantity of years equal to the life expectancy in their country minus the midpoint age of each age-stratum.

1. *Outpatient & inpatient treatment costs (direct costs to the health care system only)*
   1. **Kolkata**
      1. Outpatient cases: Sur et al 2009 [52] presented data that each outpatient case had a mean cost of $2.00 (2004 USD) with a standard deviation of $1.11. There were 67 patients in the sample, so the standard error is $0.14. After adjusting for inflation and converting to international dollars, the cost is 18.69 (SE=1.27) (2015 I$) and the corresponding distribution is Gamma(217.51, 11.64).
      2. Inpatient cases: Each inpatient case had a mean cost of $99.36 (2004 USD) with a standard deviation of $43.43.[52] There were 16 patients in the sample, so the standard error is $10.86. After adjusting for inflation and converting to international dollars, the cost is 928 (SE=101.5) (2015 $I) and the corresponding distribution is Gamma(83.75, 0.09).
   2. **Delhi**
      1. Outpatient cases: For outpatient cases, we calculate the costs as the sum of clinic visits (mean: 269 INR, SD: 394 INR), laboratory tests (mean: 157 INR, SD: 31 INR), and medicines (mean: 125 INR, SD: 86 INR) as reported by Bahl et al.[53] The sample size for all of these figures was 98 patients, so the standard errors are 39.80, 3.31, 8.68, respectively, after sampling from these distributions, adjusting for inflation and converting to international dollars, the total cost of an outpatient case is 222.12 (SE=17.48) (2015 $I). When we fit these distributions to a gamma distribution, the total cost can be describe by a distribution: Gamma(161.53, 0.73).
      2. Inpatient cases: The published data [53] on the direct cost of hospitalization borne by the health care system in Delhi is not stratified between outpatient and inpatient cases.[53] The error is evident because the total cost among the hospitalized patients was 18,131 (SD: 11,218) INR and the total costs among outpatient cases was 2,111 (SD: 1,375) INR, yet hospitalization costs were reported as only 1,316 (SD = 4,093) INR, leaving a unexplained gap of 14,704 INR in the total costs between inpatient and outpatient cases.

Therefore, we calculated the mean and standard deviation of the cost of hospitalization in the following way. The sum of the direct cost among all patients was 1,316 x 98, and the average among the 11 hospitalized patients was 11,724.36 INR. Calculating the SD among the 11 hospitalized patients was more difficult. We know that the dataset of 98 people with an SD of 4,093 contained 87 zero values and 11 non-zero values. We simulated 10,000 datasets of those non-zero values in order to find the most likely SD for the hospitalized patients, which we concluded would be 5,676 INR. We also added the cost of the clinic visit, laboratory tests, and medicines to the cost of hospitalization. After adjusting for inflation and converting to international dollars, the cost of an inpatient case is 4,840 (SE=755.20) (2015 $I) and the corresponding distribution is Gamma(41.08, 0.01).

The estimate of 11,724 INR for hospitalizations also helps explain the heterogeneity in the age-stratified costs borne by the healthcare system. These costs were reported as 663 INR for ages 0-2, which had a hospitalization rate of 0%; 2,764 INR for ages 2-5 who had a hospitalization rate of 21.4%; 1.622 for ages 5-19, who had a hospitalization rate of 8.6%; and 318 for age 19+ who had a hospitalization rate of 5.3%. If we assume that hospitalization cost only 1,316 INR, then the average costs for these groups would be approximately 551 INR, 832 INR, 664 INR, and 620 INR for each of the respective age groups, but with our estimates of outpatient/inpatient costs, our simulated estimates are closer to the observed costs: 551 INR (there were no hospitalizations among infants), 3,059 INR, 1,559 INR, and 1,172 for each of the respective age groups.

- 1. **Dong Thap:** There were no cost-of-illness data for Dong Thap, so we used estimates from a cost-of-illness study performed in Hue, Vietnam [47].
     1. Outpatient cases: Each outpatient case had a mean cost of $1.00 (2009 USD); no standard deviation was reported, so we assumed a standard error of $0.39 (assuming that the 95% confidence interval of the mean spans an interval 75% above and below the mean point estimate). After adjusting for inflation and converting to international dollars, the cost is 10.70 (SE=4.09) (2015 I$) and the corresponding distribution is Gamma(6.83, 0.64).
     2. Inpatient cases: Each inpatient case had a mean cost of $116 (2009 USD); again, no standard deviation was reported, so we assumed an SE of $44.39. After adjusting for inflation and converting to international dollars, the cost is 1,241 (SE= 475) (2015 I$) and the corresponding distribution is Gamma(6.83, 0.006).

Note: The net monetary benefits (NMB) in Dong Thap were somewhat sensitive to inpatient treatment costs (supplement Figure 12). However, directly observed characteristics in Dong Thap (moderate incidence, high probability of hospitalization) lead us to believe our conclusions are robust even to appreciable parameter uncertainty in this parameter.

- 1. **Nairobi and Lwak:** Cost data were not available for the two sites in Kenya, so we used data from a cost study conducted in Tanzania.[46] We then compared these estimates to data from a malaria cost-of-illness study in Kenya to verify that they were sensible, or at the very least conservative.[45]
     1. Outpatient cases in Tanzania: each outpatient typhoid case had a mean cost of $1.01 (2004 USD) with a standard deviation of $1.64.[46] There were 17 patients in the sample, so the standard error is $0.39. After adjusting for inflation (within Tanzania) and converting to international dollars, the cost is 4.78 (SE=1.88) (2015 I$) and the corresponding distribution is Gamma(6.45, 1.45).
     2. Inpatient cases in Tanzania: Each inpatient case had a mean cost of an outpatient case is $21.97 (2004 USD) with a standard deviation of $14.88. There were 6 patients in the sample, so the standard error is $6.07. After adjusting for inflation (within Tanzania) and converting to international dollars, the cost of an inpatient case is 104 (SE=28.72) (2015 I$) and the corresponding distribution is Gamma(13.08, 0.13).
     3. Outpatient cases in Kenya: a detailed costing study was carried out and each (uncomplicated) outpatient malaria case cost $2.82 (SE: $0.53) (USD 2007). After adjusting for inflation (within Kenya) and converting to international dollars, the cost is 17.38 (SE=3.27) (2015 I$) and the corresponding distribution is Gamma(28.31, 1.63).
     4. Inpatient cases in Kenya: Each inpatient malaria case costs 21.81 (14.59, 29.76) (USD 2007). That study presented model inputs rather than sample summaries, so the confidence interval is a standard error of the mean (rather than a standard deviation), and the SE is $3.98. After adjusting for inflation (within Kenya) and converting to international dollars, the cost is 134.40 (SE=24.50) (2015 I$) and the corresponding distribution is Gamma(30.10, 0.22).

Note: The net monetary benefits (NMB) for interventions in Kenya were not very sensitive to our assumptions about treatment costs (S12 Figure).

1. *Supply costs of vaccination*

The measure for the cost of supplies represents the cost of supplies other than the vaccine itself (i.e. syringes, cotton, alcohol). We extracted the cost estimates from the latest country Multi-Year Plans (cMYP) in each country.[42,54,55] We adjusted for inflation and converted the cost to international dollars, so the cost was 17 cents in India, 14 cents in Vietnam, and 19 cents in Kenya. Because this was an “expert” estimate rather than a sample measure, no variance was reported, so we assumed a standard error of 6 cents in India, 5 cents in Vietnam, and 7 cents in Kenya. The corresponding gamma distributions are Gamma(6.8, 40.9) in India, Gamma(6.9, 49.5) in Vietnam, and Gamma(6.9, 36.3) in Kenya.

1. *Operational costs of vaccination (cold-chain, delivery and administrative costs of vaccination)*
   1. **India:** We were unable to find survey-based estimates of the operational costs of vaccination in India, so we relied on cMYP estimates from 2012.[42] We added routine recurrent costs, routine annualized capital costs, and shared health systems costs to find the operational costs of routine vaccinations per dose administered during routine vaccination, for a total of $0.85 (USD 2012). We added campaign operational costs and shared health systems costs per dose administered during all campaigns for a total of $0.40 (USD 2012). Since no estimate of the standard error was given, we assumed that the standard error was 0.329 for routine vaccinations and 0.153 for campaigns. After adjusting for inflation and converting to international dollars, routine operational costs were $3.55 (SE: $1.36) and campaign operational costs were $1.67 (SE: $0.64) (2015 I$); the corresponding distributions were Gamma(6.83, 1.92) and Gamma(6.83, 4.08), respectively.
   2. **Vietnam:** To our knowledge, there is only one survey-based estimate of vaccine administrative costs in Vietnam (for HPV vaccination in girls), so we have applied that estimate to our model: $1.92 for routine vaccination and $2.08 for school-based campaigns (USD 2009).[44] Since no standard error was reported, we assumed standard errors of $0.73 for routine vaccination and $0.79 for campaigns. After adjusting for inflation and converting to international dollars, routine operational costs were $8.32 (SE: $3.19) and campaign operational costs were $9.02 (SE: $3.45) (2015 I$); the corresponding distributions were Gamma(6.83, 0.82) and Gamma(6.83, 0.76), respectively.

Two notes on vaccination costs in Vietnam: Although we include campaigns in our model among 5 year olds and among adults, we applied the estimate for school-based campaigns for lack of other data. It should be noted that these prices might be biased upward because HPV vaccine sessions include counseling.

- 1. **Kenya:** One survey collected data for the administrative costs of vaccination under the Expanded Programme on Immunization. To our knowledge, this is the only one survey-based estimate of vaccine administrative costs in Kenya, and that is the estimate we have used in our model: 1.33 (SD: 0.82) (USD 2012).[43] This estimate comes from four districts, so the SE is $0.205. After adjusting for inflation and converting the cost to international dollars, the cost is 3.61 (SE: 0.56) (USD 2015) and the corresponding distribution is Gamma(42.09, 11.66). No comparable estimates exist that show the difference in costs between routine vaccination and campaigns, so we have assumed that the operational costs are equivalent.

1. *Fixed parameters*
   1. Gross domestic product (GDP) per capita 2015, purchasing power parity (PPP) according to the World Bank’s World Development Indicator Database.[48] Variable name: GDP per capita, PPP (current international $).
   2. Life expectancy 2015 according to the World Bank’s World Development Indicator Database.[48] Variable name: Life expectancy at birth, total (years).
   3. We discounted costs and DALYs averted at 3% per year as per the Gates Reference Case.[36]
   4. Vaccine Wastage: We assumed a vaccine wastage rate of 15% as per WHO CHOICE recommendations.[35]
   5. Vaccine price: For scenario analysis that assessed the cost-effectiveness of interventions assuming the price of the vaccine equals the current price in India, we adjusted the price to Vietnam and Kenya using the exchange rates for USD in 2015 and converted to international dollars using the PPP conversion factor for each country in 2015 (1,800 INR = I$106 in India, I$78 in Vietnam, and I$57 in Kenya).[56]

# **Sensitivity Analyses**

To examine the robustness of our findings, we examined the relative contribution of each parameter to uncertainty in the net monetary benefits (NMB) compared to no intervention (status quo) in all sites but Delhi (where even the most ambitious intervention was unequivocally cost-saving). It is common in health economic evaluations to use statistical models to assess the importance of each uncertain input parameter on cost-effectiveness ratios (e.g. regression, ANOVA).[57] We deviated in two important ways from more traditional approaches for reasons discussed below.

First, rather than assessing the uncertainty in costs and illness separately for each intervention (amounting to twelve analyses in four sites), we assessed parameter uncertainty in the NMB of each intervention compared to no vaccination at a willingness-to-pay (WTP) threshold equal to the GDP per capita of each country (the threshold at which the WHO considers interventions “very cost-effective”). We chose to perform the analyses on NMB for three reasons: 1) because decisions are often made based on costs and effects jointly, and we aimed to assess parameter uncertainty on the most proximate index to the decision; 2) because we assessed the cost-effectiveness of multiple interventions, incremental cost-effectiveness ratios may be cumbersome to evaluate for each parameter set, especially because some of the interventions may be dominated; 3) because the difference in absolute cost-effectiveness ratios of each intervention relative to the status quo are not equal to the incremental cost-effectiveness ratios, whereas the difference in net monetary benefits relative to the comparator are equal to the incremental net monetary benefits.[57]

Second, we used random forest analysis (rather than ANOVA) to assess the relative importance of each parameter. Unlike ANOVA, random forest analysis can accommodate parameter distributions that are not normally distributed, which was true of the NMB and some of our parameter estimates. Moreover, some of the parameters are either correlated (i.e. dynamic model parameters are estimated jointly) or impact the NMB through complex relationships conditioned by other parameters (i.e. overall typhoid mortality depends on both the probability of death among hospitalized patients and the probability of hospitalization among all patients). Random forests analysis is an ensemble learning method that makes predictions based on an average set of tree models. Tree models partition the parameter space using a subsample of the variables and bootstrapped sample of the observations; the overall prediction of the random forest analysis is weighted by the error calculated with observations that were not used to estimate the tree. The error measure used to weight predictions is also the index of variable importance, which reflects the difference in error between the trees that contained the variable of interest and the trees that did not use the variable to predict the outcome (in our case, NMB).

For sensitivity analyses, we transformed the two parameters (vaccination price and number of doses required) that we had examined in scenario analyses in order to evaluate how the impact of those parameters compared to the impact of other uncertain parameters. The vaccine price was drawn from a uniform distribution ($1-$5/dose) and the number of doses was modeled as a binary variable (1 or 2 doses for children <5 years old). We implemented our analyses using the “randomForest” and the “party” packages in R.[58–61] We used the algorithm to estimate 500 trees for each “forest”; each tree was estimated using a subsample of 6 of the 19 variables, as per conventions in the literature.[61] The “party” package provides an implementation of conditional inference trees, which minimizes the bias in selecting factor parameters (e.g. the number of doses in the vaccine schedule) and parameters in complex features of the system (e.g. the probability of hospitalization and the probability of death).[60,62] We converted the absolute “mean decrease in accuracy” into a measure of “relative parameter importance”, equal to the mean decrease in accuracy of each parameter as a proportion of the sum of the mean decrease in accuracy of all parameters.

# **Additional References**

1. Pitzer VE, Bowles CC, Baker S, Kang G, Balaji V, Farrar JJ, et al. Predicting the impact of vaccination on the transmission dynamics of typhoid in South Asia: a mathematical modeling study. PLoS Negl Trop Dis. 2014;8: e2642. doi:10.1371/journal.pntd.0002642

2. Parry CM, Hien TT, Dougan G, White NJ, Farrar JJ. Typhoid fever. N Engl J Med. 2002;347: 1770–82. doi:10.1056/NEJMra020201

3. Fraser A, Paul M, Goldberg E, Acosta CJ, Leibovici L. Typhoid fever vaccines: systematic review and meta-analysis of randomised controlled trials. Vaccine. 2007;25: 7848–57. doi:10.1016/j.vaccine.2007.08.027

4. Crump JA, Mintz ED. Global Trends in Typhoid and Paratyphoid Fever. Clin Infect Dis. 2010;50: 241–6. doi:10.1086/649541

5. Hornick RB, Greisman SE, Woodward TE, DuPont HL, Dawkins AT, Snyder MJ. Typhoid fever: pathogenesis and immunologic control. N Engl J Med. 1970;283: 686–91. doi:10.1056/NEJM197010012831406

6. Ames WR, Robins M. Age and Sex as Factors in the Development of the Typhoid Carrier State, and a Method for Estimating Carrier Prevalence. Am J Public Health. 1943;33: 221–30.

7. Lanata CF, Tafur C, Benavente E L, Gotuzzo E, Carrillo C. Detection of Salmonella typhi carriers in food handlers by Vi serology in Lima, Peru. Bull Pan Am Health Organ. 1990;24: 177–182.

8. Gupta A, My Thanh NT, Olsen SJ, Sivapalasingam S, My Trinh TT, Phuong Lan NT, et al. Evaluation of community-based serologic screening for identification of chronic Salmonella typhi carriers in Vietnam. Int J Infect Dis. 2006;10: 309–14. doi:10.1016/j.ijid.2005.06.005

9. Brooks WA, Hossain A, Goswami D, Sharmeen AT, Nahar K, Alam K, et al. Bacteremic typhoid fever in children in an urban slum, Bangladesh. Emerg Infect Dis. 2005;11: 326–329. doi:10.3201/eid1102.040422

10. Naheed A, Ram PK, Brooks WA, Hossain MA, Parsons MB, Talukder KA, et al. Burden of typhoid and paratyphoid fever in a densely populated urban community, Dhaka, Bangladesh. Int J Infect Dis. International Society for Infectious Diseases; 2010;14: e93–e99. doi:http://dx.doi.org/10.1016/j.ijid.2009.11.023

11. Punjabi NH, Agtini MD, Ochiai RL, Simanjuntak CH, Lesmana M, Subekti D, et al. Enteric fever burden in North Jakarta, Indonesia: a prospective, community-based study. J Infect Dev Ctries. Italy; 2013;7: 781–787.

12. Carpenter B, Gelman A, Hoffman M, Lee D, Goodrich B, Betancourt M, et al. Stan: A Probabilistic Programming Language. J Stat Softw. 2016;VV.

13. Gelman A, Carlin JB, Stern HS, Rubin DB. Bayesian Data Analysis. 2nd ed. Boca Raton, FL USA: Chapman & Hall/CRC; 2014.

14. Sinha A, Sazawal S, Kumar R, Sood S, Reddaiah VP, Singh B, et al. Typhoid fever in children aged less than 5 years. Lancet. 1999;354: 734–7. doi:10.1016/S0140-6736(98)09001-1

15. Sur D, von Seidlein L, Manna B, Dutta S, Deb AK, Sarkar BL, et al. The malaria and typhoid fever burden in the slums of Kolkata, India: data from a prospective community-based study. Trans R Soc Trop Med Hyg. 2006;100: 725–33. doi:10.1016/j.trstmh.2005.10.019

16. Lin F-YC, Ho VA, Bay P Van, Nguyen TTT, Bryla D, Thanh TC, et al. The epidemiology of typhoid fever in the Dong Thap Province, Mekong Delta region of Vietnam. Am J Trop Med Hyg. 2000;62: 644–8.

17. Breiman RF, Cosmas L, Njuguna H, Audi A, Olack B, Ochieng JB, et al. Population-based incidence of typhoid fever in an urban informal settlement and a rural area in Kenya: implications for typhoid vaccine use in Africa. PLoS One. 2012;7: e29119. doi:10.1371/journal.pone.0029119

18. Gilman RH, Terminel M, Levine MM, Hernandez-Mendoza P, Hornick RB. Relative efficacy of blood, urine, rectal swab, bone-marrow, and rose-spot cultures for recovery of Salmonella typhi in typhoid fever. Lancet. 1975;1: 1211–3.

19. Guerra-Caceres JG, Gotuzzo-Herencia E, Crosby-Dagnino E, Miro-Quesada M, Carrillo-Parodi C. Diagnostic-Value of Bone-Marrow Culture in Typhoid-Fever. Trans R Soc Trop Med Hyg. 1979;73: 680–683. doi:10.1016/0035-9203(79)90020-8

20. Hoffman SL, Edman DC, Punjabi NH, Lesmana M, Cholid A, Sundah S, et al. Bone marrow aspirate culture superior to streptokinase clot culture and 8 ml 1:10 blood-to-broth ratio blood culture for diagnosis of typhoid fever. Am J Trop Med Hyg. 1986;35: 836–9.

21. Vallenas C, Hernandez H, Kay B, Black R, Gotuzzo E. Efficacy of bone marrow, blood, stool and duodenal contents cultures for bacteriologic confirmation of typhoid fever in children. Pediatr Infect Dis J. 1985;4: 496–8.

22. Gasem MH, Smits HL, Goris MGA, Dolmans WM V. Evaluation of a simple and rapid dipstick assay for the diagnosis of typhoid fever in Indonesia. J Med Microbiol. 2002;51: 173–177.

23. Wain J, Diep TS, Bay PVB, Walsh AL, Vinh H, Duong NM, et al. Specimens and culture media for the laboratory diagnosis of typhoid fever. J Infect Dev Ctries. 2008;2: 469–74. doi:http://dx.doi.org/10.3855/jidc.164

24. Borenstein M, Hedges L V., Higgins JPT, Rothstein HR. Introduction to Meta-Analysis. Psychother Res J Soc Psychother Res. 2009;19: 421. doi:10.1002/9780470743386

25. Gasem MH, Dolmans WM, Isbandrio BB, Wahyono H, Keuter M, Djokomoeljanto R, et al. Culture of Salmonella typhi and Salmonella paratyphi from blood and bone marrow in suspected typhoid fever. Trop Geogr Med. Amsterdam, Netherlands; 1995;47: 164–167.

26. Sur D, Ochiai RL, Bhattacharya SK, Ganduly NK, Ali M, Manna B, et al. A cluster-randomized effectiveness trial of Vi typhoid vaccine in India. N Engl J Med. 2009;361: 335–344. doi:10.1056/NEJMc091690

27. Halloran ME, Longini IM, Struchiner CJ. Design and interpretation of vaccine field studies. Epidemiol Rev. 1999;21: 73–88.

28. Lin FY, Ho HA, Khiem HB. Salmonella typhi Vi conjugate vaccine reduced the incidence of typhoid fever in 2 to 5 year old children. Evid Based Med. 2001;6: 179. doi:http://dx.doi.org/10.1136/ebm.6.6.179

29. Lanh MN, Phan VB, Ho VA, Tran CT, Lin FYC, Bryla DA, et al. Persistent efficacy of Vi conjugate vaccine against typhoid fever in young children. N Engl J Med. 2003;349: 1390–1. doi:10.1056/NEJM200310023491423

30. Mitra M, Shah N, Ghosh A, Chatterjee S, Kaur I, Bhattacharya N, et al. Efficacy and safety of vi-tetanus toxoid conjugated typhoid vaccine (PedaTyph^TM^) in Indian children: School based cluster randomized study. Hum Vaccin Immunother. 2016;12: 939–945. doi:10.1080/21645515.2015.1117715

31. Szu SC. Development of Vi conjugate - a new generation of typhoid vaccine. Expert Rev Vaccines. 2013;12: 1273–86. doi:10.1586/14760584.2013.845529

32. Mohan VK, Varanasi V, Singh A, Pasetti MF, Levine MM, Venkatesan R, et al. Safety and Immunogenicity of a Vi Polysaccharide-Tetanus Toxoid Conjugate Vaccine (Typbar-TCV) in Healthy Infants, Children, and Adults in Typhoid Endemic Areas: A Multicenter, 2-Cohort, Open-Label, Double-Blind, Randomized Controlled Phase 3 Study. Clin Infect Dis. 2015;61: 393–402. doi:10.1093/cid/civ295

33. Szu SC, Klugman KP, Hunt S. Re-examination of immune response and estimation of anti-Vi IgG protective threshold against typhoid fever-based on the efficacy trial of Vi conjugate in young children. Vaccine. Elsevier Ltd; 2014;32: 2359–63. doi:10.1016/j.vaccine.2014.02.050

34. Plummer M. JAGS: A program for analysis of Bayesian graphical models using Gibbs sampling. Proc 3rd Int Work Distrib Stat Comput. 2003; 1–10. doi:ISSN 1609-395X

35. World Health Organization. Making Choices in Health: WHO Guide to Cost-Effectiveness Analysis. Edejer TT-T, Baltussen RM, Adam T, Hutubessy R, Acharya A, Evans DB, et al., editors. Geneva, Switzerland: World Health Organization; 2002.

36. NICE International. Methods for Economic Evaluation Project: Final Report. 2014.

37. Murray CJL. Quantifying the burden of disease: The technical basis for disability-adjusted life years. Bull World Health Organ. 1994;72: 429–445. doi:10.1016/S0140-6736(96)07495-8

38. Salomon JA, Vos T, Hogan DR, Gagnon M, Naghavi M, Mokdad A, et al. Common values in assessing health outcomes from disease and injury: disability weights measurement study for the Global Burden of Disease Study 2010. Lancet. 2012;380: 2129–43. doi:10.1016/S0140-6736(12)61680-8

39. Salomon JA, Haagsma JA, Davis A, de Noordhout CM, Polinder S, Havelaar AH, et al. Disability weights for the Global Burden of Disease 2013 Study. Lancet Glob Heal. 2015;3: e712-23. doi:10.1016/S2214-109X(15)00069-8

40. Murray CJL, Vos T, Lozano R, Naghavi M, Flaxman AD, Michaud C, et al. Disability-adjusted life years (DALYs) for 291 diseases and injuries in 21 regions, 1990-2010: A systematic analysis for the Global Burden of Disease Study 2010. Lancet. 2012;380: 2197–2223. doi:10.1016/S0140-6736(12)61689-4

41. Global Burden of Disease Study 2013 Collaborators. Global, regional, and national incidence, prevalence, and years lived with disability for 301 acute and chronic diseases and injuries in 188 countries, 1990-2013: a systematic analysis for the Global Burden of Disease Study 2013. Lancet. 2015;6736: 1990–2013. doi:10.1002/14651858.CD008965.pub3.Doshi

42. Comprehensive Multiyear Plan 2013-2017: India [Internet]. [cited 29 Jun 2016]. Available: http://www.who.int/immunization/programmes_systems/financing/countries/cmyp/india/en/

43. Mvundura M, Lorenson K, Chweya A, Kigadye R, Bartholomew K, Makame M, et al. Estimating the costs of the vaccine supply chain and service delivery for selected districts in Kenya and Tanzania. Vaccine. Elsevier Ltd; 2015;33: 2697–2703. doi:10.1016/j.vaccine.2015.03.084

44. Levin CE, Van Minh H, Odaga J, Rout SS, Ngoc DNT, Menezes L, et al. Delivery cost of human papillomavirus vaccination of young adolescent girls in Peru, Uganda and Viet Nam. Bull World Health Organ. 2013;91: 585–92. doi:10.2471/BLT.12.113837

45. Conteh L, Sicuri E, Manzi F, Hutton G, Obonyo B, Tediosi F, et al. The cost-effectiveness of intermittent preventive treatment for malaria in infants in Sub-Saharan Africa. PLoS One. 2010;5. doi:10.1371/journal.pone.0010313

46. Riewpaiboon A, Piatti M, Ley B, Deen J, Thriemer K, von Seidlein L, et al. Cost of illness due to typhoid fever in Pemba, Zanzibar, East Africa. J Heal Popul Nutr. Bangladesh; 2014;32: 377–385.

47. Poulos C, Riewpaiboon A, Stewart JF, Clemens J, Guh S, Agtini M, et al. Cost of illness due to typhoid fever in five Asian countries. Trop Med Int Heal. 2011;16: 314–23. doi:10.1111/j.1365-3156.2010.02711.x

48. World Bank. World Development Indicators. Washington, DC.: World Bank (producer and distributor); 2015.

49. Bhutta ZA. Impact of age and drug resistance on mortality in typhoid fever. Arch Dis Child. 1996;75: 214–217. doi:10.1136/adc.75.3.214

50. Bhutta ZA, Naqvi SH, Durrani S, Suria A. Chloramphenicol therapy of typhoid fever. J Pak Med Assoc. 1991;41: 26–30.

51. Butler T, Islam A, Kabir I, Jones PK. Patterns of morbidity and mortality in typhoid fever dependent on age and gender: review of 552 hospitalized patients with diarrhea. Rev Infect Dis. 1991;13: 85–90.

52. Sur D, Chatterjee S, Riewpaiboon A, Manna B, Kanungo S, Bhattacharya SK. Treatment cost for typhoid fever at two hospitals in Kolkata, India. J Heal Popul Nutr. 2009;27: 725–32.

53. Bahl R, Sinha A, Poulos C, Whittington D, Sazawal S, Kumar R, et al. Costs of illness due to typhoid fever in an Indian urban slum community: implications for vaccination policy. J Heal Popul Nutr. Bangladesh; 2004;22: 304–310.

54. Comprehensive Multiyear Plan 2016-2020: Vietnam [Internet]. [cited 29 Jun 2016]. Available: http://www.who.int/immunization/programmes_systems/financing/countries/cmyp/vietnam/en/

55. Comprehensive Multiyear Plan 2011-2015: Kenya [Internet]. [cited 29 Jun 2016]. Available: http://www.who.int/immunization/programmes_systems/financing/countries/cmyp/kenya/en/

56. MedPlus Mart [Internet]. Available: http://www.medplusmart.com/product/TYPBAR-TCV-PFS-0-5ML-VACCINE/TYPB0002

57. Briggs A, Sculpher M, Claxton K. Decision modelling for health economic evaluation. Oxford University Press; 2006.

58. Liaw A, Wiener M. Classification and Regression by randomForest. R News. 2002;2: 18–22.

59. Strobl C, Boulesteix A-L, Zeileis A, Hothorn T. Bias in random forest variable importance measures: illustrations, sources and a solution. BMC Bioinformatics. 2007;8: 25. doi:10.1186/1471-2105-8-25

60. Strobl C, Boulesteix A-L, Kneib T, Augustin T, Zeileis A. Conditional variable importance for random forests. BMC Bioinformatics. 2008;9: 307. doi:10.1186/1471-2105-9-307

61. Strobl C, Hothorn T, Zeileis A. Party on! R J. 2009;1: 14–17.

62. Breiman L. Random forests. Mach Learn. 2001;45: 5–32. doi:10.1023/A:1010933404324

63. Crump JA, Luby SP, Mintz ED. The global burden of typhoid fever. Bull World Health Organ. 2004;82: 346–353.

64. Kanungo S, Dutta S, Sur D. Epidemiology of typhoid and paratyphoid fever in India. J Infect Dev Ctries. 2008;2: 454–60.

65. IndiaDemographics.com: Percentage Distribution of Population According to Age-Group and Sex in India (1992-1997) [Internet]. [cited 14 Sep 2014]. Available: http://www.indiademographics.com/demographics/7/agegroupwisepopulation/330675/agegroupwisepercentageofpopulation19712013/12977/stats.aspx

66. Demographic and Health Survey 1997: Viet Nam. Ha Noi, Vietnam; 1999.

67. Kenya National Bureau of Statistics (KNBS) and ICF Macro. Kenya Demographic and Health Survey 2008-09. Calverton, Maryland; 2010.

68. Anju S, Sunil S, Ramesh K, Seema S, Reddaiah VP, Bir S, et al. Typhoid fever in children aged less than 5 years. Lancet. 1999;354: 734–737. doi:http://dx.doi.org/10.1016/S0140-6736(98)09001-1

69. Kanungo S, Tsuzuki A, Deen JL, Lopez AL, Rajendran K, Manna B, et al. Use of verbal autopsy to determine mortality patterns in an urban slum in Kolkata, India. Bull World Health Organ. 2010;88: 667–674. doi:10.2471/BLT.09.073742

70. National Council for Population and Development. Kenya Population Situation Analysis. 2013; 12–34.

71. Feikin DR, Olack B, Bigogo GM, Audi A, Cosmas L, Aura B, et al. The burden of common infectious disease syndromes at the clinic and household level from population-based surveillance in rural and Urban Kenya. PLoS One. 2011;6: 1–10. doi:10.1371/journal.pone.0016085

72. Burton DC, Flannery B, Onyango B, Larson C, Alaii J, Zhang X, et al. Healthcare-seeking behaviour for common infectious disease-related illnesses in rural Kenya: A community-based house-to-house survey. J Heal Popul Nutr. 2011;29: 61–70. doi:10.3329/jhpn.v29i1.7567

73. Lin FY, Ho VA, Khiem HB, Trach DD, Bay P V, Thanh TC, et al. The efficacy of a Salmonella typhi Vi conjugate vaccine in two-to-five-year-old children. N Engl J Med. United States; 2001;344: 1263–1269.

74. Indian Institute of Population Science. National Family Health Survey(NFHS-II) 1998-99 Report. Mumbai, India; 2000.

75. United Nations Population Database [Internet].

76. Breiman RF, Olack B, Shultz A, Roder S, Kimani K, Feikin DR, et al. Healthcare-use for major infectious disease syndromes in an informal settlement in Nairobi, Kenya. J Heal Popul Nutr. 2011;29: 123–133. doi:10.3329/jhpn.v29i2.7854

77. Olack B, Feikin DR, Cosmas LO, Odero KO, Okoth GO, Montgomery JM, et al. Mortality trends observed in population-based surveillance of an urban slum settlement, Kibera, Kenya, 2007-2010. PLoS One. 2014;9: 2007–2010. doi:10.1371/journal.pone.0085913

78. Feikin DR, Audi A, Olack B, Bigogo GM, Polyak C, Burke H, et al. Evaluation of the optimal recall period for disease symptoms in home-based morbidity surveillance in rural and urban Kenya. Int J Epidemiol. 2010;39: 450–458. doi:10.1093/ije/dyp374

# **Supplementary Tables**

**S2 Table:** Summary of study locations.

| **Study site and dates** | **Description of surveillance** | **Clinical eligibility** | **Laboratory procedure** | **Setting description** |
| --- | --- | --- | --- | --- |
| Kolkata, India (Wards 29 and 30) **January to December 2004** [15] | Health workers visited each household each month. Practitioners from the area were encouraged to refer patients to the study health outposts. Treatment was free. | Any individual with a fever of 3 days or more. | 3-5 mL of blood was drawn from children, 8-10 mL of blood was drawn from adults.  We will assume children were <15 years of age.  Blood was incubated to allow cultures to grow, and colonies were confirmed serologically by slide and tube agglutination tests (Becton Dickinson). | A collection of legally recognized and registered slums. Extremely overcrowded, water is intermittently available through municipal taps. ‘Many’ households share municipal latrines. There is no formal sewage system.  The population was known as fairly stable [69]. Little migration has occurred in the last few decades, although individuals are known to migrate in and out occasionally. |
| Delhi, India (Kalkaji slums)  **November 1995 to October 1996**  [14] | Active surveillance was carried out: field assistants visited each household twice a week for the duration of the study. Individuals meeting the clinical eligibility criteria were referred to the study clinic for examination and blood sampling. | Blood samples were taken from children ≤5 years old if their body temperature was over 38°C. Blood samples from children >5 years of age and adults were taken if they had a continuous fever for 3 days or more. | 2 mL of blood were taken from children under the age of 5 years. 3-5 mL of blood were taken from patients over the age of 5 years. | Individuals live in “jhuggies” – structures made of clay with one or two rooms. 34% of households have piped water, and the rest receive their water from hand pumps. The sewage is “inadequate.”  Households for surveillance were chosen in clusters, rather than as one contiguous area. |
| Dong Thap Province, Viet Nam  **December 1995 to December 1996**  [16] | Provider-based passive surveillance at Commune Health Centers, Cao Lanh District Health Center, and the Provincial Hospital. Cases were confirmed by blood culture. A survey (for this study) showed that about 11% of fever cases come into contact with health services. | Fever (over 38.5°C) for more than three days. | 5 mL of blood was collected in DifcoBACTO culture bottle with 50 mL of trypticase soy broth. | The study was carried out in three communes of the Cao Lanh District of Dong Thap Province with a health center. The primary mode of transportation and source of drinking water are rivers and canals. There is no sanitation system; they use “fish-pond” latrines that are drained out to rivers. Seventy percent of households boil their water on a regular basis. |
| Nairobi, Kenya  (Kibera Slums in Nairobi Province) **March 2007 to February 2009** [17] | Home visits were performed every two weeks for consenting households.  Ill participants were encouraged to visit the field clinics, to which participants were allowed free access. Consultations, hospitalizations, and medicines were free. | Febrile illness was defined as axillary temperature ≥38.5°C until November 1, 2008 and ≥38.0°C for any duration after that | 1-3 mL of blood was collected from children ≤5 years of age and 8-10 mL of blood were collected for individuals >5 years old. Blood cultures were performed, and “standard” procedures were carried out to identify *S.* Typhi using commercial agglutinating anti-serum from Denka Seiken (Tokyo, Japan). | Africa’s largest informal settlement and the most densely populated area of Nairobi – nearly 5 times as densely populated as the city center. Land is owned by the government and people live in shacks. Only 22% of slum households in Nairobi have water connections, and 3% of households have access to public taps. Water is available at a higher-than-average price from water kiosks in the slums. A majority of residents rely on pit latrines [67,70,71]. |
| Lwak, Kenya  (Rural area in Nyanza Province)  **October 2006 to September 2009** [17] |  | Febrile illness was defined as axillary temperature ≥38.0°C for any duration |  | The catchment area spans about 100 sq. km and about 33 villages. Only 20% rural households in Kenya have improved sanitation and 54% have access to an improved source of water. The main economic activities are fishing and subsistence farming [67,72]. |

**S2 Table:** Fixed and estimated parameters of the transmission model.

| **Parameter** | **Value or Posterior Distribution^1^** | **Unit** | **Source, prior distribution** |
| --- | --- | --- | --- |
| ***Fixed transmission parameters*** | | | |
| Duration of infectiousness ***(*1/*δ)*** | 4 | weeks | [63] |
| Disease-induced mortality ***(α)*** | 0.01 | probability | [5] |
| Duration of immunity ***(*1/*ω)*** | 104 | weeks | [5] |
| ***Estimated transmission parameters*** | | | |
| Transmission rate, ***(β_0_)*** | | | |
| *Kolkata* | 0.29 (0.23, 0.35) | weekly | Uniform(0.1, 5) |
| *Delhi* | 0.40 (0.32, 0.53) | weekly |  |
| *Dong Thap* | 0.26 (0.16, 0.32) | weekly |  |
| *Nairobi* | 0.32 (0.27, 0.37) | weekly |  |
| *Lwak* | 0.17 (0.11, 0.25) | weekly |  |
| Relative transmission rate for children 0-2 years old, ***(β_1_/β_0_)*** | | | |
| *Kolkata* | 0.12 (0.02, 0.44) | fraction | Uniform(0,1) |
| *Delhi* | 0.20 (0.07, 0.55) | fraction |  |
| *Dong Thap* | 0.12 (0.003, 0.60) | fraction |  |
| *Nairobi* | 0.57 (0.19, 0.97) | fraction |  |
| *Lwak* | 0.68 (0.13, 0.98) | fraction |  |
| Relative transmission rate for children 2-5 years old ***(β_2_/β_0_)*** | | | |
| *Kolkata* | 0.29 (0.11, 0.60) | fraction | Uniform(0,1) |
| *Delhi* | 0.84 (0.51, 0.99) | fraction |  |
| *Dong Thap* | 0.60 (0.21, 0.97) | fraction |  |
| *Nairobi* | 0.95 (0.79, 1.00) | fraction |  |
| *Lwak* | 0.76 (0.26, 0.99) | fraction |  |
| Reporting rate ***(Φ_r_)*** | | | |
| *Kolkata* | 0.16 (0.12, 0.22) | probability | Uniform(0,1) |
| *Delhi* | 0.93 (0.75, 0.998) | probability |  |
| *Dong Thap* | 0.26 (0.19, 0.38) | probability |  |
| *Nairobi* | 0.29 (0.22, 0.39) | probability |  |
| *Lwak* | 0.05 (0.03, 0.11) | probability |  |
| Relative infectiousness of chronic carriers ***(r)*** | 0.35 (0.15, 0.67) | fraction | Sub-analysis, see Section S3 |
| Blood culture sensitivity  ***Φ_c_*** = 1-exp(-***ψ*** x volume of sample), | *ψ* ~ lognormal(-1.98, 0.15) | probability | Sub-analysis, see Section S2 |
| ***Demographic parameters (b)*** | | | |
| Birth rate, Urban India 2004 | 0.0115 | year^-1^ | [64] |
| Birth rate, Urban India 1995 | 0.0206 | year^-1^ | [65] |
| Birth rate, Rural Vietnam 1995 | 0.0203 | year^-1^ | [66] |
| Birth rate, Kenya Rural | 0.0325 | year^-1^ | [67] |
| Birth rate, Kenya Urban | 0.0355 | year^-1^ | [68] |

**^1^Median (95% Credible interval)**

**S3 Table:** Age-specific study data and population characteristics for Kolkata, India: January to December 2004

| Age group (in years) ^[[1]](#footnote-1),^ | Number of cases | Population | Person-years of observation | Probability of participation (***ϕ_p_***)^[[2]](#footnote-2)^ | Blood volume (mL)^[[3]](#footnote-3),^ | Mortality rate (per person per year) (***μ***) ^[[4]](#footnote-4),^ | Rate of aging (per week) | Probability of becoming a chronic carrier^[[5]](#footnote-5)^ (***θ***) |
| --- | --- | --- | --- | --- | --- | --- | --- | --- |
| <2 | 1 | 1488 | 1488 | 0.991 | 3-5 | 0.0059 | 1/104 | 0.003 |
| 2-<3 | 2 | 914 | 914 | 0.991 | 3-5 | 0.0029 | 1/52 | 0.003 |
| 3-<4 | 2 | 1034 | 1034 | 0.991 | 3-5 | 0.0020 | 1/52 | 0.003 |
| 4-<5 | 3 | 1154 | 1154 | 0.991 | 3-5 | 0.0016 | 1/52 | 0.003 |
| 5-<10 | 28 | 5609 | 5609 | 0.991 | 3-5 | 0.0010 | 1/260 | 0.003 |
| 10-<20 | 39 | 12949 | 12949 | 0.991 | 8-10 | 0.0011 | 1/520 | 0.003 |
| 20-<30 | 12 | 12049 | 12049 | 0.991 | 8-10 | 0.0016 | 1/520 | 0.021 |
| 30-<40 | 4 | 9833 | 9833 | 0.991 | 8-10 | 0.0026 | 1/520 | 0.044 |
| 40-<50 | 3 | 6994 | 6994 | 0.991 | 8-10 | 0.0052 | 1/520 | 0.088 |
| 50+ | 1 | 8448 | 8448 | 0.991 | 8-10 | 0.0479 | 0 | 0.101 |

**S4 Table:** Age-specific study data and population characteristics for Delhi, India: November 1, 1995 to October 31, 1996

| Age group | Cases^[[6]](#footnote-6),^ | Population | Person-years of observation | Mortality^[[7]](#footnote-7)^ | Weekly rate of aging | Probability of participation | Blood volume (mL) | Probability of becoming a chronic carrier^[[8]](#footnote-8)^ |
| --- | --- | --- | --- | --- | --- | --- | --- | --- |
| 0-<1 | 0 | 166 | 166 | 0.068 | 1/52 | 0.837 | 2 | 0.003 |
| 1-<2 | 5 | 202 | 202 | 0.00297 | 1/52 | 0.837 | 2 | 0.003 |
| 2-<3 | 11 | 213 | 213 | 0.00297 | 1/52 | 0.837 | 2 | 0.003 |
| 3-<4 | 5 | 225 | 225 | 0.00297 | 1/52 | 0.837 | 2 | 0.003 |
| 4-<5 | 7 | 221 | 221 | 0.00297 | 1/52 | 0.837 | 2 | 0.003 |
| 5-12 | 22 | 1579 | 1579 | 0.00115 | 1/(52*8) | 0.837 | 3-5 | 0.003 |
| 13-19 | 10 | 1164 | 1164 | 0.0018 | 1/(52*7) | 0.837 | 3-5 | 0.003 |
| 20-40 | 3 | 2684 | 2684 | 0.003 | 1/(21*52) | 0.837 | 3-5 | 0.030 |
| 40+ | 0 | 1852 | 1852 | 0.0123 | 0 | 0.837 | 3-5 | 0.101 |

**S5 Table:** Age-specific study data and population characteristics for Dong Thap Province, Vietnam: December 1995 to December 1996

| Age group | Cases^[[9]](#footnote-9),^ | Population | Person-years of observation | Mortality^[[10]](#footnote-10)^ | Weekly rate of aging | Probability of participation | Blood volume (mL) | Probability of becoming a chronic carrier^[[11]](#footnote-11)^ |
| --- | --- | --- | --- | --- | --- | --- | --- | --- |
| 0-<2 | 0 | 1190 | 1190 | 0.0067 | 1/104 | 0.421 | 5 | 0.003 |
| 2-<5 | 6 | 1785 | 1785 | 0.0033 | 1/(3*52) | 0.525 | 5 | 0.003 |
| 5-<10 | 20 | 3711 | 3711 | 0.0004 | 1/(5*52) | 0.741 | 5 | 0.003 |
| 10-<15 | 16 | 3881 | 3881 | 0.0005 | 1/(5*52) | 0.804 | 5 | 0.003 |
| 15-<20 | 4 | 2918 | 2918 | 0.0006 | 1/(10*52) | 0.804 | 5 | 0.003 |
| 20-<30 | 6 | 4278 | 4278 | 0.0007 | 1/(10*52) | 0.804 | 5 | 0.021 |
| 30-<40 | 2 | 4108 | 4108 | 0.0007 | 1/(10*52) | 0.804 | 5 | 0.044 |
| 40-<50 | 1 | 2493 | 2493 | 0.0011 | 1/(10*52) | 0.804 | 5 | 0.088 |
| 50+ | 1 | 3966 | 3966 | 0.0021 | 0 | 0.804 | 5 | 0.101 |

**S6 Table:** Age-specific study data and population characteristics for Nairobi, Kenya: March 2007 to February 2009

| Age group | Cases | Population^[[12]](#footnote-12)^ | Person-years of observation | Mortality^[[13]](#footnote-13)^ | Weekly rate of aging | Probability of participation. | Blood volume (mL) | Probability of becoming a chronic carrier^[[14]](#footnote-14)^ |
| --- | --- | --- | --- | --- | --- | --- | --- | --- |
| 0-<2 | 3 | 1,130 | 3457 | 0.0301 | 1/104 | 0.14 | 1-3 | 0.003 |
| 2-<5 | 32 | 2,703 | 6138 | 0.0086 | 1/156 | 0.41 | 1-3 | 0.003 |
| 5-<10 | 48 | 2,807 | 8049 | 0.0015 | 1/260 | 0.652 | 8-10 | 0.003 |
| 10-<18 | 24 | 4,492 | 8017 | 0.0010 | 1/416 | 0.655 | 8-10 | 0.003 |
| 18-<35 | 25 | 10,233 | 20309 | 0.0046 | 1/884 | 0.651 | 8-10 | 0.021 |
| 35-<50 | 2 | 3,530 | 6443 | 0.0096 | 1/780 | 0.8 | 8-10 | 0.044 |
| =>50 | 1 | 639 | 2120 | 0.0171 | 0 | 0.733 | 8-10 | 0.101 |

**S7 Table:** Age-specific study data and population characteristics for Lwak, Kenya: October 2006-September 2009

| Age group | Cases | Population^[[15]](#footnote-15)^ | Person-years of observation | Mortality^[[16]](#footnote-16)^ | Weekly rate of aging | Probability of participation | Blood volume (mL) | Probability of becoming a chronic carrier^[[17]](#footnote-17)^ |
| --- | --- | --- | --- | --- | --- | --- | --- | --- |
| 0-<2 | 1 | 2124 | 6544.50 | 0.0577 | 1/104 | 0.198 | 1-3 | 0.003 |
| 2-<5 | 2 | 2281 | 7027.54 | 0.0154 | 1/156 | 0.246 | 1-3 | 0.003 |
| 5-<10 | 2 | 3672 | 11312.00 | 0.0015 | 1/260 | 0.495 | 8-10 | 0.003 |
| 10-<18 | 4 | 5439 | 16756.50 | 0.0010 | 1/416 | 0.483 | 8-10 | 0.003 |
| 18-<35 | 11 | 5635 | 17359.50 | 0.0046 | 1/884 | 0.636 | 8-10 | 0.021 |
| 35-<50 | 0 | 2485 | 7655.10 | 0.0096 | 1/780 | 0.612 | 8-10 | 0.044 |
| =>50 | 2 | 3364 | 10362.00 | 0.0171 | 0 | 0.601 | 8-10 | 0.101 |

**S8 Table:** Data used to estimate vaccine parameters, from Lin et al, 2001.[73]

|  | Vaccinated Cohort | | Unvaccinated Cohort | |
| --- | --- | --- | --- | --- |
| Time period | Number of cases | Person-time | Number of cases | Person-time |
| 0-12 months | 2 | 5991*12 = 71,892 | 33 | 6017*12 = 72,204 |
| 13-27 months | 2 | (5991-2)*15 = 89,835 | 23 | (6017-33)*15 = 89,760 |
| 28-46 months | 3 | (5991-4)*19 = 113,753 | 17 | (6017-56)*19 = 113,259 |

**S9 Table.** Comparison of incremental cost-effectiveness ratios (ICER) for each of the sites for the dynamic model versus an open cohort model that only accounts for the population direct effect of vaccination (direct effect of vaccination standardized to the population [1], comparable to a static model). The shaded boxes represent the most economically viable interventions that qualify as cost-effective or better (in which the cost per DALY averted is less than three times the GDP per capita of the country).

|  | **Kolkata** | | **Delhi** | | **Dong Thap** | | **Nairobi** | | **Lwak** | |
| --- | --- | --- | --- | --- | --- | --- | --- | --- | --- | --- |
| **GDP per capita (I$, 2015)** | 6,088.60 | | | | 6,022.60 | | 3,082.50 | | | |
|  | Dynamic Model | Static Model | Dynamic Model | Static Model | Dynamic Model | Static Model | Dynamic Model | Static Model | Dynamic Model | Static Model |
| **Intervention I vs. no vaccination (status quo)** | 3,172 | 7,138 | Cost-saving | Cost-saving | Cost-saving | Cost-saving | 2,390 | 4,208 | 6,931 | 10,014 |
| **Comparison between all five interventions** | | | | | | | | | | |
| **Intervention I** | Dominated | Weakly dominated | Dominated | Dominated | Dominated | Dominated | Weakly dominated | Weakly dominated | Weakly dominated | Weakly dominated |
| **Intervention II** | Weakly Dominated | Weakly Dominated | Dominated | Dominated | Dominated | Dominated | 2,368 | 3,870 | Weakly dominated | Weakly dominated |
| **Intervention III** | 1,263 | 2,958 | Dominated | Dominated | Dominated | Cost-saving | 6,138 | 8,782 | 6,092 | 8,537 |
| **Intervention IV** | 6,238 | 12,795 | Dominated | Cost-saving | Cost-saving | 1,028 | 8,989 | 27,423 | 9,960 | 13,054 |
| **Intervention V** | 20,442 | 96,510 | Cost-saving | 27,456 | 1,655 | 10,895 | 23,628 | 193,425 | 17,007 | 34,979 |

# **Supplementary Figures**

**S1 Figure** Dynamic model of disease transmission and vaccination. Black boxes correspond to the infection process in the absence of vaccination and red boxes correspond to vaccinated states. Orange lines depict the infection process, blue lines depict the recovery process, green lines depict the process by which individuals become chronic carriers, purple lines depict waning immunity, either natural immunity or vaccine-conferred immunity, and red lines signify the vaccination process. Red dashed lines represent vaccination on individuals who would not benefit from the vaccine. Model parameters describe the time-varying rates at which the transitions between states occur.

**S2 Figure:** Compartmental model depicting the process of vaccination, waning immunity, and infection in the randomized-control trial of Vi-rEPA typhoid conjugate vaccination.


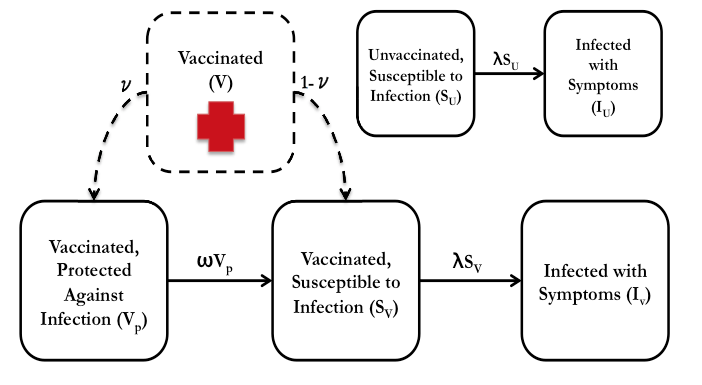

**S3 Figure:** Relationship between blood volume and culture sensitivity for the detection of typhoid fever. The circles represent the mean and error bars show the 95% credible intervals for the blood culture sensitivity estimated among bone-marrow culture-positive cases in eight studies. The grey shaded regions correspond to model predictions.

**S4 Figure:** Relationship between time since vaccination and protection. Markers represent observed cumulative protection in the Vi-rEPA randomized control trial and the error bars show the 95% confidence intervals of the estimates. A) The model-predicted vaccine efficacy over time for a sample of 100 pairs of values of the initial protection and the rate of waning immunity drawn from the joint posterior distribution. B) The model-predicted median protection for all iterations is represented by the dark gray line, while the 95% credible interval is shown shaded in light gray.

**S5 Figure:** Observed and model-predicted incidence of typhoid fever across five sites. The black bars show the incidence observed in population-based studies and the light gray bars show the incidence predicted by the model (including both the transmission and the observation process). The error bars show the 95% confidence intervals for incidence (corresponding to the black bars) and the 95% credible intervals of the model-predicted incidence.

**S6 Figure:** Model-predicted incidence of typhoid fever in all age groups after TCV introduction. Coverage is assumed to be 80% for routine vaccination and 70% for one-time campaigns. The colored lines represent the mean model predictions, while the shaded regions represent the corresponding 95% prediction intervals.

**
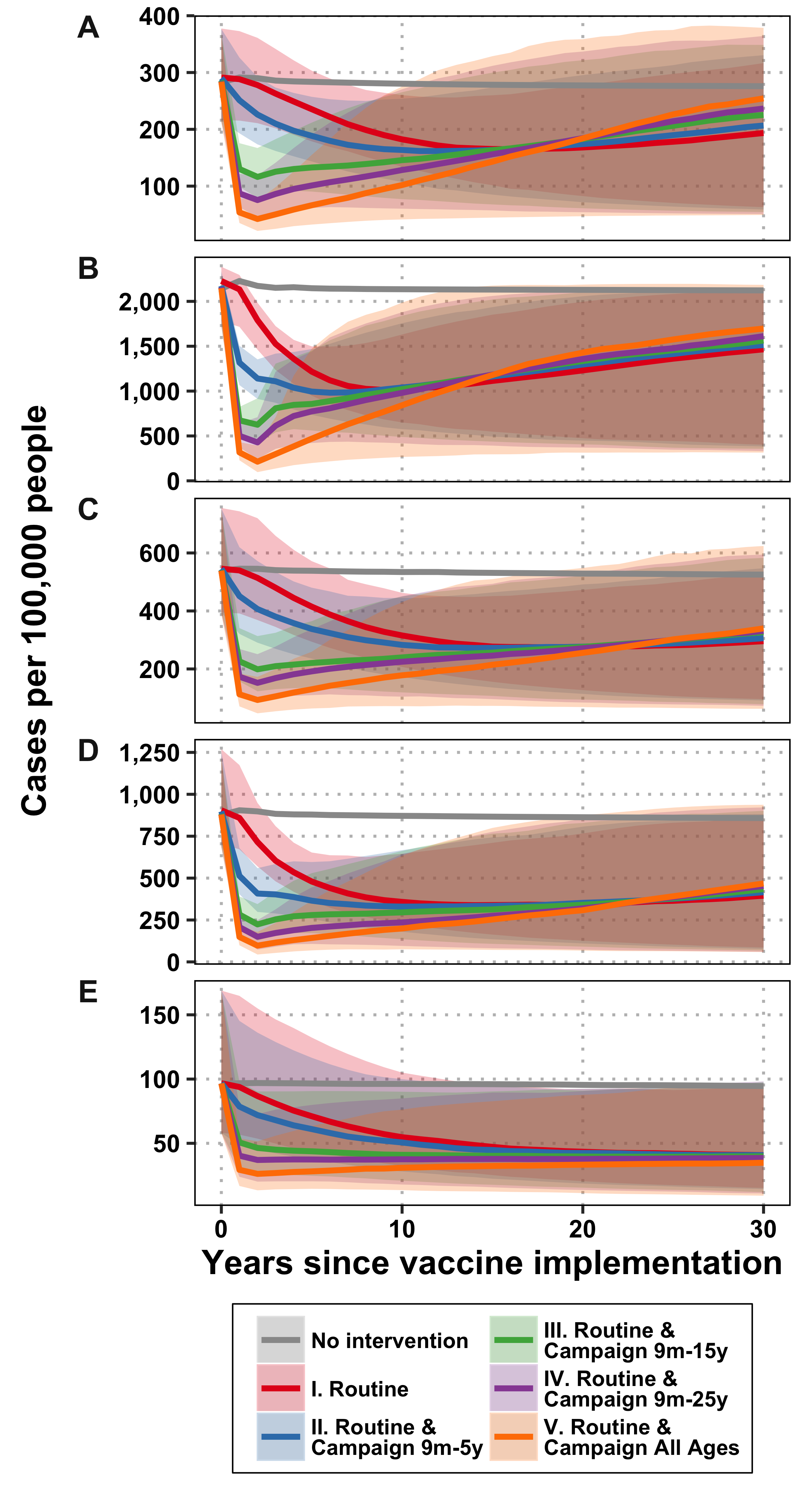
**

**S7 Figure.** Probability that routine vaccination is cost-effective compared to no intervention. The stacked bars represent the probability that routine vaccination meets the threshold for being cost-saving, very cost-effective (cost per disability-adjusted life years (DALYs) averted less than per capita gross domestic product (GDP)), cost-effective (cost per DALYs averted less than three times GDP), or not cost-effective (cost per DALYs averted greater than three times GDP) in each setting, given uncertainty in the model parameters.

**S8 Figure:** The impact of the price per dose of the vaccine on cost-effectiveness of routine vaccination under the assumption that immunization requires a one-dose schedule. Cost-effectiveness acceptability curves for routine vaccination at 9 months of age are shown for (A) Kolkata, (B) Delhi, (C) Dong Thap, (D) Nairobi, and (E) Lwak. The dotted line shows the threshold at which an intervention is considered cost-saving, while the dashed line delineates the threshold at which an intervention is considered very cost-effective and the dot-dashed line delineates the threshold at which an intervention is considered cost-effective by the WHO criteria in each country.

**S9 Figure:** The impact of the price per dose of the vaccine on cost-effectiveness of routine vaccination under the assumption that immunization requires a two-dose schedule for children <5 years of age. Cost-effectiveness acceptability curves for routine vaccination at 9 months of age are shown for (A) Kolkata, (B) Delhi, (C) Dong Thap, (D) Nairobi, and (E) Lwak. The dotted line shows the threshold at which an intervention is considered cost-saving, while the dashed line delineates the threshold at which an intervention is considered very cost-effective and the dot-dashed line delineates the threshold at which an intervention is considered cost-effective by the WHO criteria in each country.

**S10 Figure:** The impact of price of vaccination on cost-effectiveness of all delivery strategies under the assumption that immunization requires a one-dose schedule. Cost-effectiveness acceptability curves for all five delivery strategies are shown for (A) Kolkata, (B) Delhi, (C) Dong Thap, (D) Nairobi, and (E) Lwak. The dotted line shows the threshold at which an intervention is considered cost-saving, while the dashed line delineates the threshold at which an intervention is considered very cost-effective and the dot-dashed line delineates the threshold at which an intervention is considered cost-effective by the WHO criteria in each country.

**S11 Figure:** The impact of the price per dose of the vaccine on cost-effectiveness of all delivery strategies under the assumption that immunization requires a two-dose schedule for children <5 years of age and a one-dose schedule for all others. Cost-effectiveness acceptability curves for all five delivery strategies are shown for (A) Kolkata, (B) Delhi, (C) Dong Thap, (D) Nairobi, and (E) Lwak. The dotted line shows the threshold at which an intervention is considered cost-saving, while the dashed line delineates the threshold at which an intervention is considered very cost-effective and the dot-dashed line delineates the threshold at which an intervention is considered cost-effective by the WHO criteria in each country.

**S12 Figure:** Impact of uncertainty in each parameter on the net monetary benefits (NMB) of each intervention as compared to the status quo, estimated using random forest analysis. Results are shown for (A) Kolkata, (B) Delhi, (C) Dong Thap, (D) Nairobi, and (E) Lwak. The transmission parameters are a) transmission rate, b) transmission rate for ages <2, c) transmission rate for ages 2-5, d) contribution of chronic carriers, e) probability reporting to clinic. The treatment outcome parameters are f) probability of hospitalization, g) probability of death, h) Years lost to disability (YLD): outpatient case, i) YLD: inpatient case, j) YLD weight. Treatment cost parameters are: k) outpatient costs, l) inpatient costs. Parameters regarding vaccine characteristics: m) probability vaccine protection, n) waning rate, o) number of doses. Parameters regarding vaccine costs are: p) vaccine price, q) vaccine supply costs, r) operational costs of routine vaccination, s) operational costs of vaccine campaigns.

**S13 Figure**: The difference in impact as predicted by a dynamic model versus a static model of typhoid incidence. We compared our dynamic model output, which accounts for the reduction in incidence to due both the direct protection of vaccinated individuals as well as the indirect protection of unvaccinated individuals through a reduction in the force of infection, to the expected reduction in incidence due to the direct effect of vaccination alone (population direct effect).[1] Impact was denominated as cases averted over 10 years since vaccine introduction.

**S14 Figure:** Cost-effectiveness of a one-dose schedule at the current market price of the Typbar-TCV vaccine in India (1800 INR; Bharat Pharmaceuticals). Cost-effectiveness acceptability curves for routine vaccination at 9 months of age (left) and for all five delivery strategies under consideration (right) versus no intervention for (A) Kolkata, (B) Delhi, (C) Dong Thap, (D) Nairobi, and (E) Lwak. The dotted line shows the threshold at which an intervention is considered cost-saving, while the dashed line delineates the threshold at which an intervention is considered very cost-effective and the dot-dashed line delineates the threshold at which an intervention is considered cost-effective by the WHO criteria in each country.

**S15 Figure:** Cost-effectiveness of a one-dose schedule at I$1 per dose in a scenario where the transmission parameter decreases by 50% over 30 years due to improvements in sanitation. Cost-effectiveness acceptability curves for routine vaccination at 9 months of age (left) and for all five delivery strategies under consideration (right) versus no intervention for (A) Kolkata, (B) Delhi, (C) Dong Thap, (D) Nairobi, and (E) Lwak. The dotted line shows the threshold at which an intervention is considered cost-saving, while the dashed line delineates the threshold at which an intervention is considered very cost-effective and the dot-dashed line delineates the threshold at which an intervention is considered cost-effective by the WHO criteria in each country.

1. Age groups were top-coded at age 50, the last two age groups (60-70 and 70+) had no cases. [↑](#footnote-ref-1)
2. The incidence study [15] reported 3683 fever visits, 47 of which were repeat, follow- up visits, which we excluded from our calculations. 31 visits took place where no blood culture was performed. Therefore, the probability of blood culture was (3683-47-31)/(3683-47) = 0.991. [↑](#footnote-ref-2)
3. The incidence study [15] states that 3-5 mL of blood were taken from children and 8-10 mL of blood were taken from adults; we designated the cut-off to be 10 years of age. [↑](#footnote-ref-3)
4. Mortality data was retrieved from a demographic study of the study catchment population [69]. A spline was fit to estimate the rate of death for intermediate age groups for which there was no discrete mortality data. Mortality for 2-5 year olds was reported to be 0.00241, in the paper.[69] [↑](#footnote-ref-4)
5. These estimate come from Ames et al, 1943.[6] [↑](#footnote-ref-5)
6. The incidence study only surveyed the population under 40 years old.[14] We assumed there were 0 cases in ages 40 years and over. To calculate the total incidence, we added the person-time for people over age 40 years, which we determined to be 22.3% of the total population.[65] [↑](#footnote-ref-6)
7. Yearly mortality for children between 1 and 5 years of age was calculated from the national infant and under 5 mortality rate reported in the 1998-9 DHS Final Report: -log(1-(95-68)/1000)/4. Other mortality rate data was retrieved from the age-specific urban mortality rate from the 1998-9 DHS Final Report.[74] [↑](#footnote-ref-7)
8. These estimate come from Ames et al, 1943.[6] [↑](#footnote-ref-8)
9. The number of cases was back-calculated from the published incidence rates. The incidence study reported a total of 28,239 people living in this area; we applied the rural population age distribution from the DHS 1997 Report for Vietnam.[66] [↑](#footnote-ref-9)
10. Mortality data was obtained from the UN population statistics database.[75] [↑](#footnote-ref-10)
11. These estimate come from Ames et al, 1943.[6] [↑](#footnote-ref-11)
12. Population from a survey in July 2005 on health care seeking [76]; there is a need to re-calculate these because of different age-cutoffs. The health-care use survey covers Gatwikira and Soweto, the two villages in the surveillance programs in 2007-2010. Population 5-<10: 8,422(5/15). Population 10-<18: 8,422(8/15). Population 18-35: 8,422(2/15) +7,185 +3,850/2. Population for 35-<50: 3,850/2 + 1,605. [↑](#footnote-ref-12)
13. This was mortality for Kibera for January 2007-December 2010. It was necessary to recalculate the figures for children <5 due to different age-cutoffs in both studies. <1 mortality is actually 50.2 per 1,000 PYO, <2 mortality: -log((1000-50.2)*(1-8.6/1000)/1000)/2. Mortality for 2-<5 has stayed the same.[77] [↑](#footnote-ref-13)
14. These estimates come from Ames et al, 1943.[6] [↑](#footnote-ref-14)
15. According to Feiken 2010[78], there were 25,000 people in this survey; we multiplied 25,000 times the proportion of the total person-years of observation attributable to each group. [↑](#footnote-ref-15)
16. From DHS 2008-09.[67] Mortality 2-5 population: -log((1000-149)/(1000-95))/4. Under 2: -log((1000-95)*(1-under_2_mort)/1000)/2. [↑](#footnote-ref-16)
17. These estimate come from Ames et al, 1943.[6] [↑](#footnote-ref-17)
